# An intelligent tribological hydrogel delivering 9,10-DiOH activates cuproptosis for comprehensive rheumatoid arthritis intervention

**DOI:** 10.1016/j.mtbio.2026.103502

**Published:** 2026-07-29

**Authors:** Yuntao Li, Hongji Liu, Fangzhou Xie, Hanwen Chang, Yiwei Zhang, Yuxin Zhang, Yu Fu, Wenjun Tang, Zengguang Wang, Hairong Tao, Xiuping Cheng

**Affiliations:** a Department of Traditional Chinese Medicine, The First Affiliated Hospital of Wannan Medical University(Yijishan Hospital of Wannan Medical University), Wuhu, 241000, China; b Department of Orthopedics, Shanghai Key Laboratory of Orthopedic Implant, Shanghai Ninth People's Hospital, Shanghai Jiao Tong University School of Medicine, Shanghai, 200011, China; c School of Physical Science and Technology, Shanghai Tech University, Shanghai, 201210, China; d State Key Laboratory of Chemical Biology, Shanghai Institute of Organic Chemistry, Chinese Academy of Sciences, Shanghai, 200032, China

**Keywords:** Rheumatoid arthritis, Cuproptosis, 9,10-Dihydroxyoctadecanoic acid, Ferredoxin 1, Stimuli-responsive hydrogel

## Abstract

Rheumatoid arthritis (RA) is a debilitating autoimmune disease characterized by persistent synovial hyperplasia, severe oxidative stress, and progressive biomechanical destruction of articular cartilage. Current pharmacological interventions predominantly rely on systemic immunosuppression, which fails to address localized mechanical wear and suffers from rapid intra-articular clearance and off-target toxicity. Herein, we present a unified biological and materials-science strategy by engineering an intelligent, stimuli-responsive hydrogel composite (CS-RB/DiOH) that simultaneously restores joint tribology and modulates lipid metabolism. Through comprehensive chemical profiling, we identified 9,10-dihydroxyoctadecanoic acid (9,10-DiOH) as a core bioactive lipid mediator. Integrated multi-omics analyses reveal that 9,10-DiOH engages ferredoxin 1 (FDX1)-dependent cuproptosis, thereby suppressing NF-kB and MAPK inflammatory signaling, inhibiting osteoclastogenesis, and attenuating synovial fibroblast hyperproliferation. To overcome delivery barriers, 9,10-DiOH is conjugated into a chitosan-based polymeric network via dynamic covalent boronate ester bonds. This architecture enables RGD-mediated active targeting and temperature-, reactive oxygen species (ROS)-, and pH-triggered precision drug release. Crucially, the exposure of the lipid's hydrophobic alkyl chains at the interface provides robust biomimetic boundary lubrication, reducing the intra-articular coefficient of friction. In a murine RA model, this localized depot effectively alleviates synovitis, attenuates bone erosion, and helps preserve joint architecture. This work highlights a mechanism-driven therapeutic paradigm that synergizes metabolic cuproptosis induction with tribological restoration for comprehensive RA management.

## Graphical abstract

Lipid-derived 9,10-dihydroxyoctadecanoic acid (9,10-DiOH) activates FDX1-dependent cuproptosis, thereby suppressing osteoclastogenesis and inflammatory signaling through the MAPK/NF-kB pathways. A temperature-, ROS-, and pH-responsive tribological hydrogel further enables targeted local delivery, enhancing anti-inflammatory efficacy and joint repair in rheumatoid arthritis.

**Figure:**
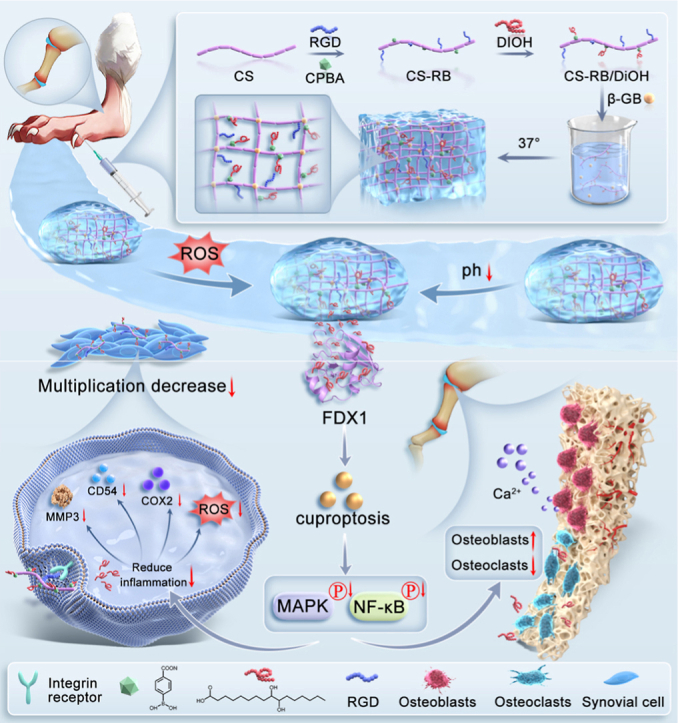

## 1 Introduction

Rheumatoid arthritis (RA) is a chronic autoimmune disease characterized by persistent synovial inflammation, progressive joint destruction, and systemic immune dysregulation [[1], [2], [3], [4]]. A central pathological feature of the disease is aberrant synovial hyperplasia, which drives cartilage degradation and irreversible bone erosion, ultimately leading to joint deformity and functional disability [[5], [6], [7]]. Among the various cellular components within the synovial microenvironment, fibroblast-like synoviocytes play a pivotal role in disease progression. These cells undergo pathological activation and acquire aggressive, tumor-like phenotypes, including sustained proliferation, resistance to apoptosis, and excessive secretion of pro-inflammatory cytokines and matrix-degrading enzymes. Such alterations not only perpetuate synovial inflammation but also promote osteoclast activation and bone destruction. Although current therapeutic strategies primarily rely on immunosuppressive agents and biologics, these approaches are often limited by systemic side effects, treatment resistance, and high economic burden, underscoring the need for alternative strategies that target disease-driving cellular and metabolic mechanisms [[8], [9], [10]].

Accumulating clinical and epidemiological evidence indicates that fatty acids exert protective effects against the development of autoimmune diseases, including rheumatoid arthritis [11,12]. Dietary supplementation with specific fatty acids has been associated with a significantly reduced incidence of rheumatoid arthritis and attenuation of disease severity, suggesting that lipid metabolism represents an underexplored regulatory axis in autoimmune pathology. However, despite these compelling clinical observations, the molecular mechanisms underlying the anti-arthritic effects of fatty acids remain poorly understood. In particular, how lipid-derived molecules influence synovial fibroblast behavior, inflammatory signaling [13,14], and joint-destructive pathways has not been systematically investigated, limiting their translational application in rheumatoid arthritis therapy.

Cuproptosis is a copper-dependent form of regulated cell death that is closely associated with mitochondrial metabolism. Mechanistically, intracellular copper accumulation promotes the interaction between copper and lipoylated tricarboxylic acid cycle-related proteins, resulting in mitochondrial proteotoxic stress and disruption of energy metabolism. FDX1 has been identified as an upstream regulator of this process by modulating copper redox cycling and the accumulation of lipoylated mitochondrial proteins. Given that RA progression involves mitochondrial metabolic remodeling, oxidative stress, inflammatory signaling, and osteoclast-mediated bone erosion, FDX1-associated copper-dependent regulation may provide a mechanistic link between metabolic stress and inflammatory joint destruction.

Despite the compelling therapeutic promise of lipid-derived molecules, their clinical translation is severely hindered by inherent physicochemical limitations. Systemic administration often leads to off-target effects and suboptimal drug accumulation in inflamed joints. While intra-articular injection offers localized intervention, small-molecule drugs are typically subjected to rapid lymphatic clearance, resulting in a transient therapeutic window. Furthermore, the RA joint microenvironment is highly complex; it is characterized not only by hyperplastic fibroblast-like synoviocytes (FLSs) and severe oxidative stress but also by mechanically compromised, friction-burdened articular cartilage. Conventional drug delivery systems lack the active targeting capability to selectively penetrate pathological FLSs and fail to provide the essential biomechanical lubrication needed to mitigate progressive cartilage wear. Therefore, the development of an intelligent delivery platform that integrates active cellular targeting, disease-microenvironment responsiveness, and biomimetic tribological restoration is highly desired but remains a formidable challenge.

Herein, we present a unified biological and materials-science strategy to overcome these barriers. Through comprehensive chemical profiling of lipid-rich traditional formulations, we first identify 9,10-DiOH as a shared core lipid mediator. Integrated multi-omics analyses reveal that 9,10-DiOH activates a ferredoxin 1 (FDX1)-dependent cuproptosis pathway, which suppresses NF-kB and MAPK signaling, attenuates osteoclastogenesis, and restrains the pathological proliferation of FLSs. To bridge the translational gap, a multifunctional, stimuli-responsive chitosan-based hydrogel composite (CS-RB/DiOH) is engineered. This system is rationally designed with RGD peptides for active FLS targeting and utilizes dynamic covalent boronate ester bonds to enable temperature-, reactive oxygen species (ROS)-, and pH-triggered sustained release of 9,10-DiOH. Notably, the incorporation of the long alkyl chains from 9,10-DiOH provides robust boundary lubrication, reducing the friction coefficient of the damaged joint. Together, these findings establish a mechanism-driven lipid metabolism-based therapeutic strategy and provide an integrated biomaterial-based delivery platform for RA intervention [15].

## 2 Methods

### 2.1 Animals, ethics approval, and CIA induction

Male SPF-grade DBA/1 J mice (8 weeks old, 20-25 g; Shanghai Jihui Laboratory Animal Care Co., Ltd) were housed under controlled conditions (22 ± 2°C, 50 ± 5% humidity, 12-h light/dark cycle) with ad libitum access to standard chow and sterile water and were acclimatized for 1 week before experiments. All animal experiments were approved by the Experimental Animal Welfare and Ethics Committee of Wannan Medical College (Approval No. LLSC-2022-242). Collagen-induced arthritis (CIA) was induced by emulsifying bovine type II collagen (CII, 2 mg/mL) with complete Freund's adjuvant (CFA, 4 mg/mL) to form a stable emulsion, followed by intradermal injection at the base of the tail for primary immunization (day 0). A booster immunization was administered on day 21 using CII (2 mg/mL) emulsified with incomplete Freund's adjuvant (IFA). Prominent arthritis signs typically appeared around day 28, and mice were then enrolled for therapeutic interventions.

Randomization was performed using a computer-generated randomization procedure. To minimize potential cage effects, mice in each group were distributed across two independent cages (2 + 3 per cage). The right hind paw/ankle was predefined as the primary endpoint for clinical assessment. Clinical arthritis scoring and right ankle swelling measurements (digital caliper) were performed independently by two blinded investigators, and the summed scores were used for statistical analysis. Prespecified exclusion criteria included failure to develop arthritis by day 28 (no swelling and clinical score = 0), severe procedure-related adverse events (e.g., aspiration during gavage or technical failure of intra-articular injection), severe infection/unexpected death, or meeting humane endpoints (e.g., >15–20% body weight loss, severe immobility, or ulceration). No animals were excluded and no dropouts occurred in the present study (allocated n = analyzed n).

### 2.2 *In vivo* oil-intervention study

For the oil-intervention study, CIA mice were randomized into four groups (n = 5 per group): Normal control, CIA model, CIA + Linwa oil (petroleum ether fraction), and CIA + peach kernel (petroleum ether fraction). The petroleum ether fractions were prepared as described in the extraction subsection and suspended in 2% (v/v) Tween-80 to a working concentration of 0.2 g/mL (crude drug equivalent). Treatments were initiated after arthritis onset (day 28). Mice received oral gavage every other day from day 28 to day 49 at a crude drug equivalent dose of 1 g/kg with a fixed dosing volume of 5 mL/kg, while the Normal control and CIA model groups received an equal volume of 2% Tween-80 on the same schedule. Clinical arthritis scoring and right ankle swelling (primary endpoint) were assessed every 3 days from day 28 to day 49. Mice were euthanized by CO_2_ inhalation on day 49, and ankle joints and major organs were collected for downstream analyses (e.g., micro-CT, histology, TRAP staining, immunostaining, and biosafety evaluations).

### 2.3 *In vivo* biomaterial study

An independent cohort of CIA mice was used for the biomaterial study to avoid cross-interference with the oil-intervention experiment. Mice were randomized into five groups (n = 5 per group): Normal, CIA, CIA + blank CS-RB hydrogel, CIA + free 9,10-DiOH, and CIA + CS-RB/DiOH hydrogel. To minimize procedure-related inflammation caused by repeated puncture, intra-articular injections were performed once weekly on days 28, 35, and 42 after primary immunization (total 3 injections). All injections were administered unilaterally into the right ankle (tibiotalar) joint at a fixed volume of 8 μL per mouse per injection. The Normal and CIA groups received PBS (vehicle), the blank-hydrogel group received CS-RB hydrogel precursor without DiOH, the free-DiOH group received 9,10-DiOH at 100 μM (31.6 μg/mL; 0.253 μg per injection), and the CS-RB/DiOH hydrogel group received CS-RB/DiOH hydrogel precursor loaded to the same nominal DiOH concentration (100 μM; 0.253 μg per injection). Clinical arthritis scoring and right ankle swelling (primary endpoint) were assessed every 3 days from day 28 to day 49. Mice were euthanized by CO_2_ inhalation on day 49, and the injected right ankle joints and major organs were harvested for subsequent analyses.

### 2.4 Analysis of drug components

Sample preparation and LC-MS/MS analysis were performed by OE Biotech Co., Ltd. (Shanghai, China). Briefly, 100 mg of sample was extracted with 1 mL of methanol (containing 2 μg/mL internal standards) via grinding at 60 Hz (−40°C) and ultrasonication for 60 min. After centrifugation (12,000 rpm, 4°C, 10 min), the supernatant was analyzed using an ACQUITY UPLC I-Class system (Waters) coupled with a Thermo-Orbitrap-QE HF mass spectrometer. Separation was achieved on an ACQUITY UPLC HSS T3 column (100 mm × 2.1 mm, 1.8 μm) at 45°C, using a gradient of water (0.1% formic acid) and acetonitrile at 0.35 mL/min. Mass spectrometry was operated in HESI mode with DDA acquisition (Full MS/dd-MS2, TOP 10). Compounds were identified by matching accurate mass (error <5 ppm), MS2 fragments, and retention times against the LuMet-TCM database and supplemented by the Herb database. 9,10-Dihydroxyoctadecanoic acid, also known as 9,10-dihydroxystearic acid, was purchased from Bidepharm Co., Ltd. (Shanghai, China; Cat. No. BD111569; CAS No. 120-87-6; molecular formula: C18H36O4; molecular weight: 316.48; purity: 97%).

### 2.5 Cell culture and in vitro inflammation model

Cell culture and TNF-α-induced inflammatory activation. Human fibroblast-like synoviocytes derived from rheumatoid arthritis synovial tissue (HFLS-RA; Guangzhou Genio Biotech, China; biosafety level 1) were cultured according to the supplier's instructions. Cells were maintained in high-glucose DMEM (4.5 g/L glucose) supplemented with 15% fetal bovine serum (FBS) in a humidified incubator at 37°C with 5% CO_2_. The culture medium was renewed twice per week. For passaging, the monolayer was briefly rinsed (<2 min) with 0.25% trypsin–0.53 mM EDTA to remove residual serum, followed by additional trypsin–EDTA digestion at room temperature for ≤60 s; digestion was immediately neutralized with complete medium and cells were gently dissociated and reseeded at 3–5 × 10^3^ cells/cm^2^. To mimic an RA-relevant inflammatory microenvironment, HFLS-RA cells were stimulated with TNF-α (10 ng/mL) for 24 h prior to subsequent treatments or analyses. Successful inflammatory activation was verified by increased expression and/or secretion of representative pro-inflammatory mediators (IL-6, IL-1β, TNF-α), upregulation of the surface marker CD54, and activation of canonical inflammatory signaling (e.g., NF-kB/MAPK phosphorylation), as assessed by qPCR, Western blotting, and flow cytometry.

### 2.6 Live/dead staining assay

Cells were washed thoroughly 2-3 times with 1× PBS to remove residual esterase activity. Following PBS removal, adherent cells were incubated with a sufficient volume of Calcein AM/PI staining working solution (prepared per manufacturer's instructions of the Live/Dead Fixable Cell Staining Kit) (PF00007, Proteintech, China) at room temperature, protected from light, for 15-20 min. Fluorescent microscopy was subsequently employed to visualize the stained cells.

### 2.7 EdU proliferation assay

Cell proliferation was assessed using the EdU (5-ethynyl-2′-deoxyuridine) incorporation assay according to the manufacturer's instructions. Briefly, cells were seeded in 6-well plates and treated as previously described. The cells were then incubated with 20 μM EdU working solution for 2 h at 37°C. After labeling, the culture medium was removed, and the cells were fixed with 1 mL of fixative solution for 15 min at room temperature. Subsequently, the cells were washed three times (5 min each) with washing buffer, followed by incubation with 1 mL of permeabilization buffer for 10 min at room temperature to facilitate antibody penetration. After two additional washes, the cells were incubated with the Click-it reaction cocktail for 30 min at room temperature in the dark. Finally, the cell nuclei were counterstained with Hoechst 33342 after three washes. The percentage of EdU-positive cells was visualized and analyzed using a fluorescence microscope.

### 2.8 Detection of intracellular reactive oxygen species

Intracellular Reactive Oxygen Species (ROS) levels were measured using the fluorescent probe DCFH-DA (2′,7′-dichlorodihydrofluorescein diacetate). Briefly, cells were seeded in 6-well plates as previously described. The DCFH-DA probe was diluted in PBS to a final concentration of 10 μM, and 1 mL of the dilution was added to each well. The plates were then incubated at 37°C in a 5% CO_2_ incubator for 20 min in the dark. After incubation, the cells were washed three times with PBS (5 min each) to remove the extracellular probe. The fluorescence intensity, representing the ROS levels, was immediately visualized and captured using a laser scanning confocal microscope (LSCM).

### 2.9 Quantification of labile Cu by fluorescent dye CF4

The cells were first washed with PBS for excess medium, and then 0.8 μm CF-4 was added And incubated at 37°C and 5% CO_2_ for 10 min. After culture, cells were washed twice with phenol red free MEM to remove excess probe, then stained with DAPI for 10 min, then washed and stored in phenol red free MEM at 37°C and 5% CO_2_ for another 20 min. Developed with a confocal microscope.

### 2.10 Immunofluorescence

Following fixation in ice-cold 4% paraformaldehyde (15 min) and permeabilization using 0.5% Triton X-100 (5 min), cells were blocked with immunostaining blocking buffer (Beyotime, Shanghai, China; 1 h). Subsequently, samples were exposed to primary antibodies targeting overnight at 4°C. After three TBST washes the next day, cells were treated with FITC/Cy3-conjugated anti-rabbit secondary antibody (Proteintech, Wuhan, China; 1 h, room temperature). Nuclei were visualized by counterstaining with 4’,6-diamidino-2-phenylindole (DAPI) working solution (Beyotime, Shanghai, China; 10 min). Imaging was performed using either a Leica confocal laser scanning microscope or a DM4000 B epifluorescence microscope (Leica Microsystems GmbH, Wetzlar, Germany).

### 2.11 Micro-CT

We employed a SkyScan 1276 Micro-CT system for scanning and subsequent three-dimensional (3D) reconstruction. Image analysis focused on a region of interest (ROI) spanning slices 10 to 110, using the growth plate slice (designated as 0 mm) as the reference point. Grayscale contrast was set within the range of 68 to 255. Using CTAn software (Bruker micro CT), we performed 3D analysis, bone mineral density (BMD),bone surface to bone volume ratio (BS/BV), trabecular number (Tb.N) assessment, and generated 3D models, which were subsequently modified in CT Vox software (Bruker micro CT).

### 2.12 TRAP staining

For the induction of osteoclasts, bone marrow-derived macrophages (BMMs) were obtained from bone marrow stromal cells (BMSCs) extracted using the aforementioned primary cell isolation protocol. The cells were cultured in induction medium supplemented with 50 ng/mL M-CSF (macrophage colony-stimulating factor) for 7 days. Subsequently, the cells were seeded into 24-well plates and further stimulated with 20 ng/mL RANKL (receptor activator of nuclear factor-kappa B ligand) for an additional 7 days to promote osteoclast differentiation. After the induction period, tartrate-resistant acid phosphatase (TRAP) activity was assessed using a TRAP/ALP Stain Kit (Wako, Japan) following the manufacturer's instructions. TRAP-positive cells containing three or more nuclei were identified and photographed under a light microscope.

### 2.13 RNA‐Sequencing

Transcriptome sequencing and analysis were outsourced to MetWare Biotechnology Co., Ltd. Total RNA isolation utilized TRIzol reagent (Ambion; Thermo Fisher Scientific, MA, USA), with the resulting RNA adjusted to a concentration equivalent to 3 μg for subsequent library construction. Libraries were prepared according to the manufacturer's instructions using the Illumina®-compatible NEBNext® Ultra™ RNA Library Prep Kit (NEB, USA), incorporating unique index codes for sample multiplexing. Following library preparation, fragments within the target size range (250-300 bp) were isolated using the AMPure XP system (Beckman Coulter, Beverly, USA). Library quality control was performed on the Bioanalyzer 2100 system. Finally, the double-stranded cDNA libraries underwent sequencing on Illumina platforms. Raw RNA-seq reads were comprehensively analyzed: HISAT2 v2.0 aligned reads to the reference genome, followed by transcript assembly with StringTie (v1.3.3 b) guided by the reference. Gene expression quantification utilized featureCounts v1.5.0-p3. Statistical significance was assessed with the Benjamini-Hochberg procedure for false discovery rate (FDR) control, yielding adjusted P-values. Differentially expressed genes (DEGs) were defined as those exhibiting an adjusted P-value ≤0.05 and an absolute log2 fold change (|log2 FC|) ≥ 0.5.

### 2.14 RT-qPCR

RNA was extracted from cultured cells using the AxyPrep Multisource Total RNA Miniprep Kit (Axygen, Corning, NY, USA). The procedure involved lysing cells, followed by sequential centrifugation and washing steps with the kit's provided buffers. The resulting RNA pellet was resuspended in TE buffer and centrifuged to obtain purified RNA. Complementary DNA (cDNA) synthesis was then performed via reverse transcription using the TaKaRa Reverse Transcription Kit (TaKaRa, Shiga, Japan). RT-qPCR analysis was subsequently carried out on the QuantStudio 6 Flex Real-Time PCR System (Applied Biosystems, CA, USA), employing SYBR Green PCR Master Mix (Bimake, TX, USA).

### 2.15 Western blot

Cellular proteins were lysed in RIPA buffer (P0013B, Beyotime, China) containing protease and phosphatase inhibitors (78445, Thermo Scientific, USA). Following sonication, protein concentration was determined using a BCA assay. Equal amounts of protein (20 μg per lane) underwent electrophoresis and were transferred to 0.22 μm PVDF membranes (Millipore, USA). The membranes were blocked with 5% BSA, then incubated with primary antibodies overnight at 4°C. After TBST washes, secondary antibody incubation proceeded for 2 h at 4°C. Protein bands were finally detected using a chemiluminescence imaging system (Touch Imager, e-BLOT, China). Antibodies used: MMP3 (82984-4-RR, proteintech, China), COX2 (12375-1-AP, proteintech, China), SLC31A1(67221-1-Ig, proteintech, China), COX IV (ab16056, abcam, US), TOMM20 (ab186735, abcam, US), p38MAPK (80821-2-RR, proteintech, China), phospho-p-38 MAPK (81212-2-RR, proteintech, China), NF-kB p65 (80979-1-RR, proteintech, China), Phospho-NF-kB p65 (82335-1-RR, proteintech, China), DLAT (13426-1-AP, proteintech, China), FDX1 (12592-1-AP, proteintech, China), IkB (10268-1-AP, proteintech, China), Phospho-IkB(82349-1-RR, proteintech, China), Phospho-ERK1/2(28733-1-AP,proteintech, China), ERK1/2(11257-1-AP,proteintech, China), NFATc1 (cat. No. ab2796; mouse mAb), c-Fos (cat. No. 9F6; rabbit mAb), CTSK (cat. No. E7U5N; rabbit mAb), Beta Actin(66009-1-Ig, proteintech, China), Protein marker (P9006, NCM, China)

### 2.16 Immunofluorescence and cytoskeleton staining

Following fixation in ice-cold 4% paraformaldehyde (15 min) and permeabilization using 0.5% Triton X-100 (5 min), cells were blocked with immunostaining blocking buffer (Beyotime, Shanghai, China) for 1 h at room temperature. Subsequently, the cells were incubated with Copper Fluor-4 primary antibody (dilution as per manufacturer's instructions) overnight at 4°C. After three washes with PBS the following day, the cells were treated with FITC or Cy3-conjugated anti-rabbit secondary antibody (Proteintech, Wuhan, China) for 1 h at room temperature in the dark. To visualize the actin cytoskeleton, the cells were then incubated with Phalloidin (Beyotime, Shanghai, China) for 1 h. After two additional washes with PBS, nuclei were counterstained with 4′,6-diamidino-2-phenylindole (DAPI) working solution (Beyotime, Shanghai, China) for 10 min. Fluorescence imaging was performed using either a Leica confocal laser scanning microscope or a DM4000 B epifluorescence microscope (Leica Microsystems GmbH, Wetzlar, Germany).

### 2.17 Flow cytometric analysis

FLSs were seeded into 6-well plates and assigned to five groups: Control, LPS, 9,10-DiOH, siFDX1, inhibition. Following the respective treatments, cells were dissociated using trypsin and collected by centrifugation. The cell pellets were then resuspended in ice-cold PBS for washing. To evaluate the expression of surface markers, the cells were incubated with a specific antibody against CD54 (ICAM-1) in the dark, according to the manufacturer's instructions. For each sample, at least 10,000 events were acquired using a flow cytometer. Data were analyzed using FlowJo software (or specified version) to determine the percentage of CD54-positive cells and the mean fluorescence intensity (MFI)

### 2.18 Cell transfection

Four siRNAs targeting FDX-1 were sourced from Generalbiol Co., Ltd. (Anhui, China). Cells were plated in 6-well plates and allowed to reach 60-80% confluence prior to transfection. Following the manufacturer's protocol (Thermo Fisher Scientific, USA), transfection was carried out using Lipofectamine™ RNAiMAX Transfection Reagent. Specifically, each siRNA (10 μmol/L) was diluted in Opti-MEM Reduced-Serum Medium (Gibco, Thermo Fisher Scientific, USA). Equal volumes of the diluted siRNA solution and the transfection reagent were combined, incubated at room temperature for 5 min, and then applied to the cells. The efficiency of gene knockdown was subsequently confirmed via Western blotting analysis.

### 2.19 CCK‐8

Cells were plated in 96-well plates at an exact density of 5 × 10^3^ cells per well to ensure uniform distribution and optimal growth. This density was chosen to achieve sufficient coverage while preventing overcrowding. After a 12/24/48-h incubation period to allow for adaptation, adherence, and early growth initiation, cell proliferation was evaluated using the CCK-8 assay (Sigma-Aldrich, St. Louis, MO, USA). Absorbance readings at 450 nm, reflecting metabolic activity and proliferation, were obtained using an Infinite M200 Pro microplate reader (Tecan Group Ltd., Männedorf, Switzerland). Experimental groups consisted of a control and concentrations of 0.1, 1, and 10 μmol/ml.

### 2.20 Preparation and chemical characterization of CS-RB/DiOH

Chitosan (CS; degree of deacetylation ∼80-90%) (1.00 g) was dissolved in 100 mL of aqueous acetic acid (1% v/v) under magnetic stirring at room temperature overnight. The pH of the CS solution was then adjusted to 5.5 by dropwise addition of 1.0 M NaOH, and the system was buffered with 0.1 M MES buffer (pH 5.5). To confer active targeting capability, the RGD peptide was first conjugated to the primary amine groups of CS. Briefly, the carboxyl groups of the RGD peptide were activated using EDC·HCl and NHS chemistry in MES buffer for 30 min, and then reacted with the CS solution under continuous stirring for 24 h at room temperature. The mixture was extensively dialyzed (MWCO 3.5 kDa) against deionized water and lyophilized to obtain RGD-functionalized chitosan (denoted as CS-R).

Next, to introduce the reactive oxygen species (ROS) and pH-responsive moieties, 4-carboxyphenylboronic acid (CPBA) (0.50 g, 3.0 mmol) was activated by EDCHCl (0.69 g) and NHS (0.41 g) in 30 mL of 0.1 M MES (pH 5.5) for 30 min. The activated CPBA solution was then added dropwise to the CS-R solution. The reaction was allowed to proceed under stirring for 12 h at room temperature. The resulting mixture was purified by dialysis (MWCO 3.5 kDa) against deionized water for 72 h, followed by lyophilization to synthesize the boronic-acid-functionalized targeted polymer (denoted as CS-RB).

For drug loading, CS-RB (100 mg) was dissolved in 10 mL of borate buffer (0.05 M, pH 8.5). 9,10-DiOH was then added at a 1:1 M ratio relative to the boronic acid moieties on CS-RB. The mixture was stirred for 2 h at room temperature to promote dynamic covalent boronate ester formation between the vicinal diols of the drug and the boronic acid groups, yielding the final targeted and drug-loaded composite, CS-RB/DiOH.

The step-by-step synthesis and chemical structures were confirmed by ^1^H NMR and FTIR spectroscopy. In the 1H NMR spectrum, CS-R exhibited characteristic resonance peaks corresponding to the specific amino acid residues of the RGD peptide compared with pristine CS. Following CPBA conjugation, CS-RB showed newly appeared aromatic proton signals at 7-8 ppm, indicating successful grafting of phenylboronic acid moieties. After conjugation with 9,10-DiOH, additional aliphatic resonances attributable to the long alkyl chain (0.8-2.5 ppm) became evident in CS-RB/DiOH. FTIR further verified the synthesis: CS-R and CS-RB exhibited progressively strengthened amide-related bands (1650 and 1550 cm^−1^) consistent with successive amide bond formations, while CS-RB/DiOH displayed enhanced aliphatic C-H stretching bands (2920/2850 cm^−1^) together with changes in the B-O/C-O region (1350-1100 cm^−1^), confirming the boronate ester interactions.

### 2.21 Preparation and performance evaluation of CS-RB/DiOH hydrogels

CS-RB/DiOH was dissolved in aqueous acetic acid (1% v/v) to prepare a 2.0 wt% polymer solution (200 mg CS-B/DiOH in 10 mL of acetic acid solution), stirred magnetically at 4°C overnight until completely dissolved, and then pre-cooled for subsequent use. β-Glycerophosphate (β-glycerophosphate, β-GP; sodium salt) was prepared as a 50 wt% stock solution (5.0 g β-GP dissolved in deionized water and brought to a final volume of 10 mL) and likewise pre-cooled at 4°C. For thermosensitive hydrogel formation, the pre-cooled β-GP stock solution was slowly added dropwise to the CS-B/DiOH solution under an ice bath with gentle mixing at a volume ratio of CS-B/DiOH solution to β-GP (50 wt%) of 1.00 mL:0.30 mL. The resulting homogeneous mixture remained flowable and injectable at low temperature and was immediately incubated at 37°C (incubator or water bath) to induce thermogelation and form the thermosensitive hydrogel.

Rheological measurements of the hydrogel were performed using an HR-10 rheometer (TA Instruments, New Castle, DE, USA) equipped with 40 mm parallel plates. The sample was loaded with an appropriate gap. An amplitude (strain) sweep was conducted at 37°C at a fixed angular frequency of 10 rad/s over a strain range of 0.001-1000%. Temperature-ramp tests were carried out under oscillatory conditions at a constant heating rate of 2 °C/min; the temperature was increased from 10 to 40°C for the 1:0.1 formulation, while a 10-30°C ramp was used for the 1:0.3 and 1:0.5 formulations. The gelation temperature was determined as the crossover point where the storage modulus (G′) equaled the loss modulus (G″). The reversible thermally triggered sol-gel transition was further evaluated by cyclic temperature switching between 4 and 37°C (4°C-37°C, 4°C-37°C), during which G′ and G″ were continuously recorded. In addition, injectability (shear-thinning behavior) was assessed by a shear-rate sweep from 0.1 to 10 s^−1^.

A calibration curve for DiOH quantification was established using PBS-based standard solutions(10-300 ng/mL). Samples were analyzed on an SCIEX QTRAP 4500 liquid chromatography–tandem mass spectrometry (LC-MS/MS) system, and the chromatographic and mass spectrometric parameters were optimized. Linear regression was obtained by plotting peak area versus concentration for the standards (R^2^ > 0.99), and the resulting equation was used to determine DiOH concentrations in release samples. For the in vitro release study, 2 mL of the drug-loaded hydrogel was sealed in a dialysis bag (MWCO = 1 kDa) and immersed in 30 mL of release medium under continuous gentle stirring at 37°C. To evaluate stimulus responsiveness, three media were employed: PBS at pH 7.4, an acidic buffer at pH 5.5, and an ROS-mimicking medium prepared by adding H_2_O_2_ (final concentration, 1 mM) to PBS (pH 7.4). At predetermined time intervals, 1 mL of the external medium was withdrawn and immediately replaced with an equal volume of fresh corresponding medium. The amount of released DiOH was quantified by LC-MS/MS. All experiments were performed in triplicate, and data are presented as mean ± standard deviation.To determine the total drug loading, 2 μL of freshly prepared CS-RB/DiOH hydrogel (or 2 mg lyophilized sample) was completely disrupted in 1 mL of 0.1 M NaOH with sonication for 30 min, diluted to 2 mL, and quantified by LC-MS/MS (n = 3).Additional release media were included to mimic the joint microenvironment: simulated synovial fluid (SSF: pH 7.4 PBS containing 2% BSA and 0.3% HA) and enzymatic degradation medium (pH 7.4 PBS containing hyaluronidase 1500 U mL^−1^). All other procedures were the same as described above.

### 2.22 Friction coefficient test of hydrogels

The frictional coefficient of the hydrogels was determined using a friction and wear testing machine (MFT-R4000).To evaluate the progressive enhancement in lubricating efficacy conferred by the sequential chemical modifications, the fabricated CS, CS-R, CS-RB, and CS-RB/DiOH hydrogels (formulated at a fixed optimal β-GP ratio of 1:0.5) were systematically tested.Furthermore,to investigate the impact of the thermosensitive crosslinking density on the tribological performance,the final CS-RB/DiOH hydrogels were prepared with varying ratios of β-GP (1:0.3,1:0.5,and 1:0.7).

During the test, the hydrogel samples were affixed to a polyethylene surface to ensure stability during shear. A steel ball was employed as the upper counter-frictional pair to slide against the hydrogel surface. We tracked the correlation between the coefficient of friction (COF) and time throughout the experiment. To simulate the dynamic mechanical stress of articular joints, the operational parameters were set as follows: the frictional normal load was applied at 1 N, the setting amplitude was 5 mm, the reciprocating sliding frequency was 1 Hz, and the duration of each test was 20 min. All tests were performed at a physiological temperature of 37°C. The average COF values were calculated from the steady-state regions for subsequent statistical analysis.

### 2.23 Statistical analysis

Results are expressed as mean ± SD (n = 3 or 5 per group, derived from independent experiments or repeated measurements). All statistical analyses were performed using GraphPad Prism software (version 8.0). Differences between two groups were analyzed using an unpaired two-tailed Student's t-test. For comparisons among three or more groups, one-way analysis of variance (ANOVA) followed by Tukey's post hoc test was performed. For time-course experiments involving multiple groups and time points, data were analyzed using two-way ANOVA followed by Bonferroni's multiple comparisons test. A p-value of <0.05 was considered statistically significant.

## 3 Results

### 3.1 Lipid-based treatment alleviates inflammation and osteoclast-mediated bone destruction in CIA mice

**Fig. 1:**
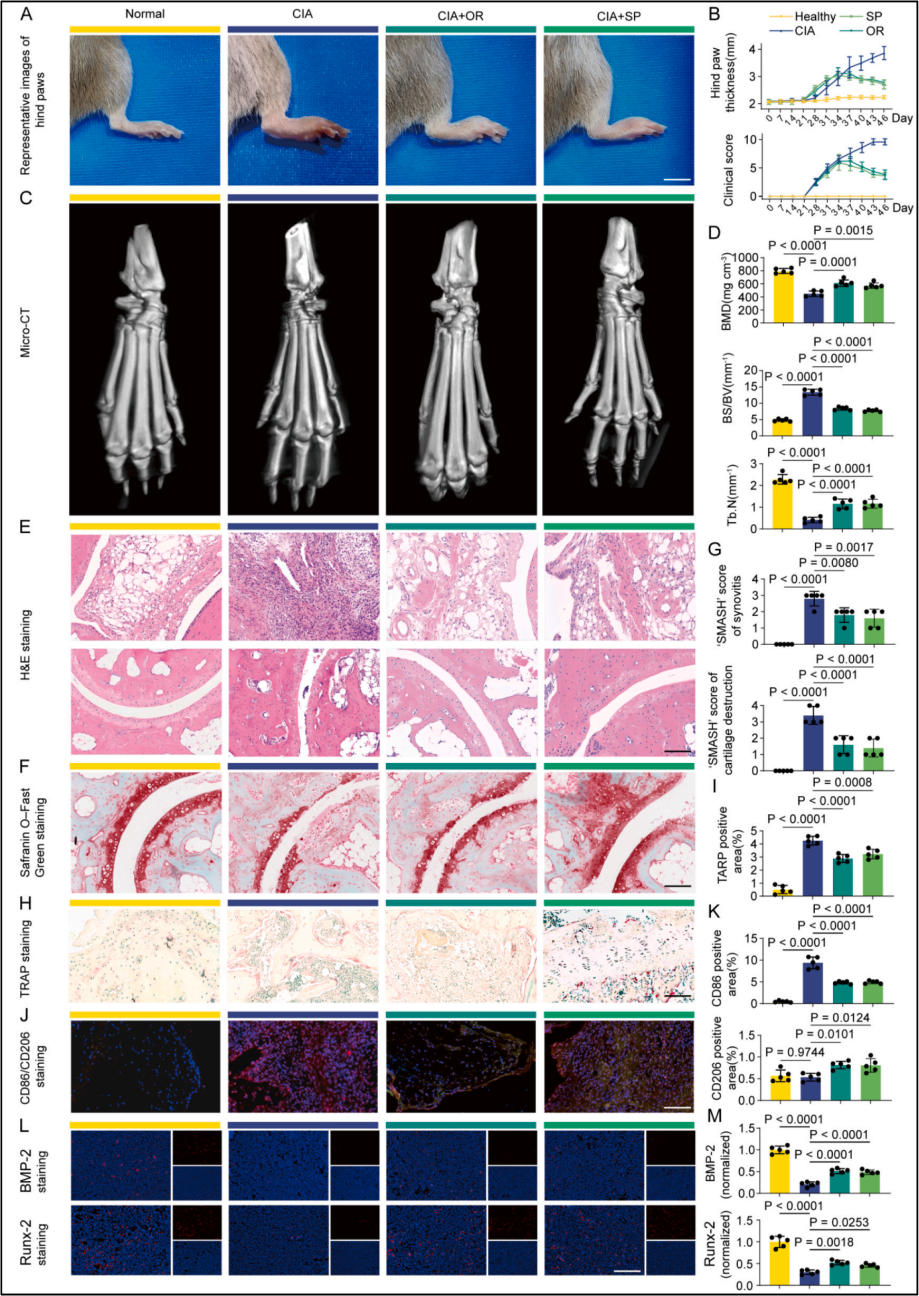
**Multimodal assessment of joint inflammation and bone remodeling following lipid-based treatment in CIA mice** (**A)** Representative gross images of hind paws from normal, CIA, animal oil–treated (OR), and plant oil-treated (SP) groups (scale bar = 500 μm). **(B)** Quantification of hind paw thickness and clinical arthritis scores during disease progression. **(C)** Representative Micro-CT images of hind limb joints. **(D)** Quantitative analysis of bone parameters including BMD, BS/BV, and Tb.N. **(E)** Hematoxylin and eosin (H&E) staining of joint sections showing synovial inflammation. **(F)** Safranin O-Fast Green staining assessing cartilage integrity. **(G)** Semi-quantitative scores of synovitis and cartilage destruction. **(H)** TRAP staining indicating osteoclast activity. **(I)** Quantification of TRAP-positive area. **(J)** CD86 and CD206 staining of joint sections. **(K)** Quantitative analysis of CD86^−^and CD206-positive areas. **(L)** Representative fluorescence images of osteogenic markers BMP-2 and Runx-2. (M) Quantitative analysis of BMP-2 and Runx-2 fluorescence intensity (scale bar = 50 μm). (For interpretation of the references to color in this figure legend, the reader is referred to the Web version of this article.)

Lipid-based interventions markedly attenuated inflammation and osteoclast-mediated bone destruction in CIA mice. Compared with untreated CIA mice, administration of animal-derived (Oviductus Ranae) or plant-derived (Persicae Semen) lipids significantly reduced hind paw swelling and clinical arthritis scores, as demonstrated by representative images and quantitative analyses (Fig. 1A and B). Micro-computed tomography revealed substantial preservation of bone architecture following lipid treatment accompanied by significant improvements in bone mineral density, bone surface to bone volume ratio, and trabecular number (Fig. 1C and D). Histological examination further showed reduced synovial hyperplasia and inflammatory infiltration (Fig. 1E), along with marked protection of articular cartilage integrity as evidenced by Safranin O-Fast Green staining (Fig. 1F). Consistently, tartrate-resistant acid phosphatase staining demonstrated a pronounced reduction in osteoclast number and activity (Fig. 1H and I), indicating suppression of bone resorption. Immunostaining analysis revealed decreased expression of the pro-inflammatory macrophage marker CD86 and increased expression of the anti-inflammatory marker CD206 (Fig. 1J and K), suggesting remodeling of the synovial immune microenvironment. In parallel, fluorescence staining of osteogenic markers showed significant upregulation of BMP-2 and Runx-2 following lipid treatment (Fig. 1L and M), indicating enhanced osteogenic potential. To identify the bioactive components underlying these effects, compositional analysis of both lipid preparations was performed, leading to the identification of 9,10-DiOH as a shared core component selected for subsequent mechanistic investigations (Supplementary Fig. S1 + Supplementary Tables S1–S3).

### 3.2 Metabolomic profiling reveals pronounced mitochondrial dysfunction induced by 9,10-DiOH

**Fig. 2:**
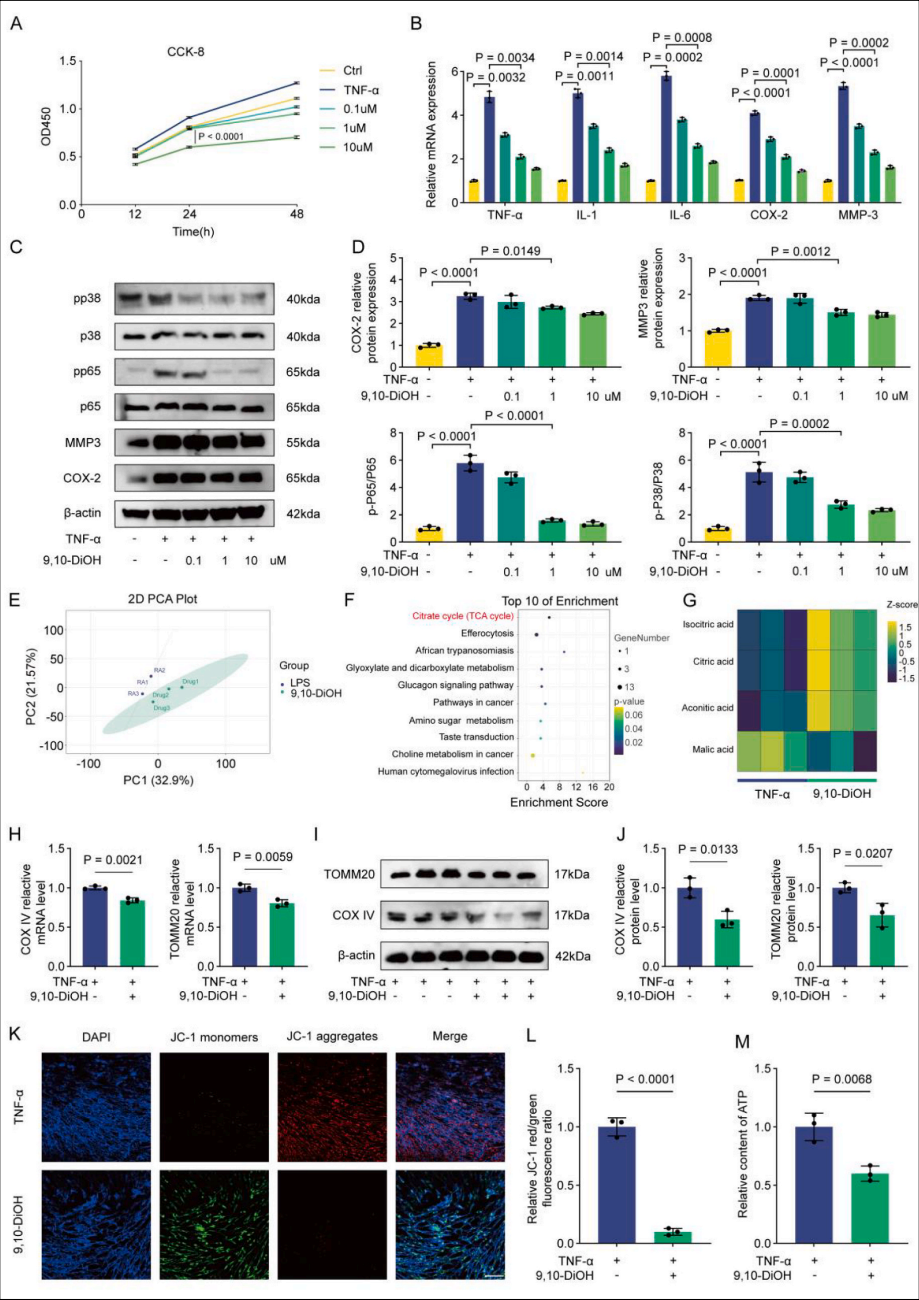
**9,10-DiOH suppresses inflammatory signaling and disrupts mitochondrial function *in vitr***o. (**A)** Cell viability following treatment with increasing concentrations of 9,10-DiOH was assessed by CCK-8 assay at 12, 24, and 48 h. **(B)** Relative mRNA expression levels of tumor necrosis factor-α, interleukin-1β, interleukin-6, cyclooxygenase-2, and matrix metalloproteinase-3 were determined by quantitative PCR. **(C**–**D)** Representative western blot images showing phosphorylated and total p38 and p65, as well as cyclooxygenase-2 and matrix metalloproteinase-3 protein levels, with corresponding quantitative analyses. Cells were treated with 0, 0.1, 1, and 10 μM 9,10-DiOH for 24 h. **(E)** Principal component analysis of metabolomic profiles illustrating metabolic differences between groups. **(F)** Top enriched metabolic pathways identified by pathway enrichment analysis. **(G)** Heatmap visualization of key metabolites involved in the tricarboxylic acid cycle. **(H**–**J)** Relative mRNA and protein expression levels of cytochrome *c* oxidase subunit IV and translocase of outer mitochondrial membrane 20 following 9,10-DiOH treatment. **(K)** Representative images of JC-1 staining. Red fluorescence indicates JC-1 aggregates in polarized mitochondria, while green fluorescence indicates JC-1 monomers in depolarized mitochondria (Scale bar: 50 μm). **(L)** Quantification of JC-1 red/green fluorescence ratio. Data are expressed relative to TNF-α group. **(M)** Intracellular ATP levels measured by luminescence assay. Values normalized to TNF-α group. Data are shown as mean ± SEM (n = 3). (For interpretation of the references to color in this figure legend, the reader is referred to the Web version of this article.)

To determine the optimal working concentration of the lipid-derived compound, cell viability was first assessed using a CCK-8 assay, which identified 1 μM at 24 h as the most suitable concentration for subsequent experiments (Fig. 2A). Quantitative PCR analysis demonstrated that treatment with 9,10-DiOH markedly suppressed the transcription of key pro-inflammatory mediators, including tumor necrosis factor-α, interleukin-1β, interleukin-6, cyclooxygenase-2, and matrix metalloproteinase-3 (Fig. 2B). Consistently, western blot analysis revealed a pronounced reduction in the phosphorylation levels of p38 and p65, accompanied by decreased protein expression of cyclooxygenase-2 and matrix metalloproteinase-3 (Fig. 2C and D), indicating the dual inhibition of inflammatory and osteoclastogenic signaling pathways. To further elucidate the underlying metabolic alterations, untargeted metabolomic profiling was performed in vitro, revealing a clear separation between groups in principal component analysis (Fig. 2E). Pathway enrichment analysis identified the tricarboxylic acid cycle as one of the most significantly upregulated pathways (Fig. 2F), with several key metabolites exhibiting marked changes (Fig. 2G), suggesting a profound impact on mitochondrial metabolism. Immunofluorescence staining of mitochondrial markers further demonstrated a substantial reduction in cytochrome *c* oxidase subunit IV and translocase of outer mitochondrial membrane 20 signals following 9,10-DiOH treatment (Supplementary Fig. S2). These observations were corroborated by quantitative PCR and western blot analyses, which confirmed significant downregulation of cytochrome *c* oxidase subunit IV and translocase of outer mitochondrial membrane 20 at both the transcriptional and protein levels (Fig. 2H–J). Collectively, these data indicate that 9,10-DiOH induces pronounced mitochondrial dysfunction, which may contribute to its anti-inflammatory and anti-osteoclastogenic effects in fibroblast-like synoviocytes. Furthermore, mitochondrial membrane potential was evaluated using JC-1 staining. Compared with the TNF-α group, 9,10-DiOH treatment markedly decreased JC-1 aggregate fluorescence and increased JC-1 monomer fluorescence, resulting in a significantly reduced red/green fluorescence ratio (Fig. 2K and L). Consistently, intracellular ATP levels were also significantly decreased after 9,10-DiOH treatment (Fig. 2M). These functional mitochondrial assays further confirmed that 9,10-DiOH impaired mitochondrial activity and energy metabolism in TNF-α-stimulated fibroblast-like synoviocytes.

### 3.3 Integrated transcriptomic, proteomic, and functional analyses support FDX1-associated copper-dependent regulation by 9,10-DiOH

**Fig. 3:**
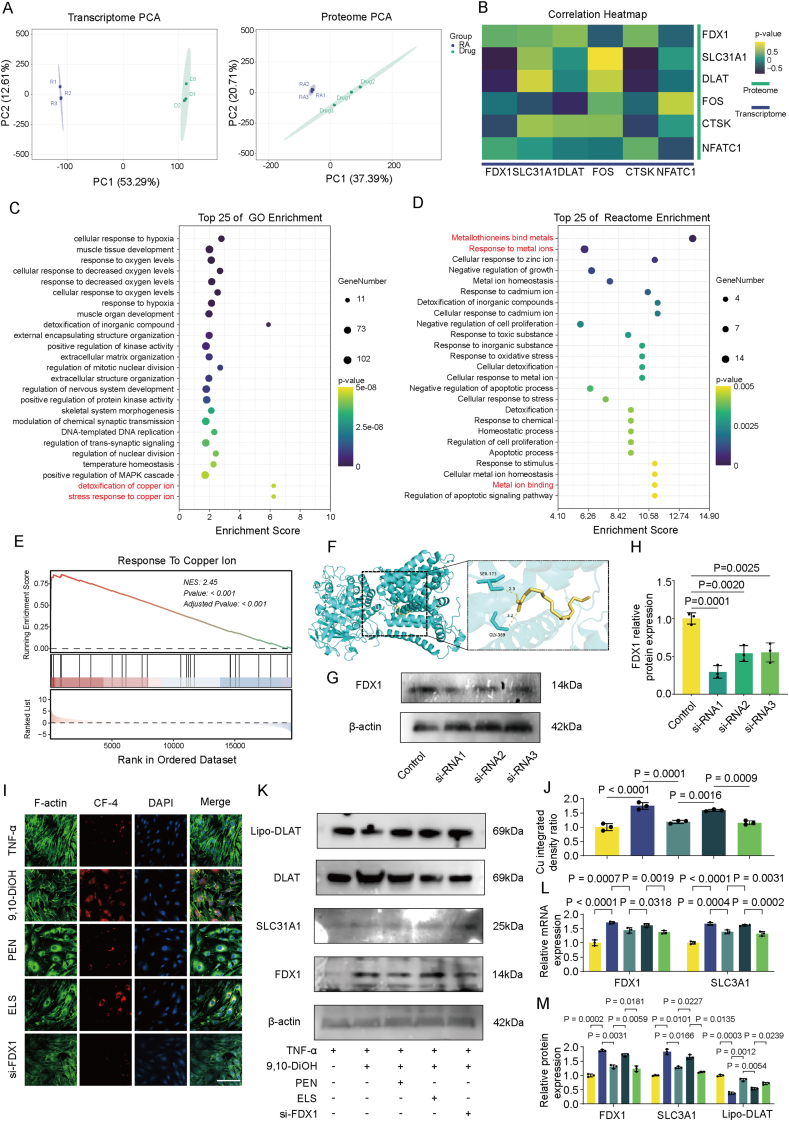
**9,10-DiOH activates FDX1-dependent cuproptosis through coordinated transcriptomic and proteomic regulatio**n. (A) Principal component analysis of transcriptomic and proteomic datasets following 9,10-DiOH treatment. **(B)** Integrated correlation analysis between transcriptomic and proteomic data highlighting key cuproptosis-associated genes. **(C**–**D)** Top 25 enriched Gene Ontology biological processes and Reactome pathways. **(E)** Gene set enrichment analysis showing activation of the cellular response to copper ions. **(F)** Molecular docking simulation illustrating the interaction between 9,10-DiOH and ferredoxin 1. **(G**–**H)** Validation of ferredoxin 1 knockdown efficiency by western blot analysis, with corresponding quantitative analysis. **(I)** Immunofluorescence analysis of intracellular copper levels under different treatment conditions (scale bar = 50 μm). **(J)** Relative mRNA expression levels of ferredoxin 1, solute carrier family 3 member 1, and dihydrolipoamide S-acetyltransferase determined by quantitative PCR. **(K**–**L)** Representative western blot images showing expression of ferredoxin 1, solute carrier family 3 member 1, and lipoylated dihydrolipoamide S-acetyltransferase, with quantitative analysis of protein expression levels.

To further elucidate the molecular mechanism underlying the biological effects of 9,10-DiOH, integrated transcriptomic and proteomic analyses were performed. Principal component analysis demonstrated a clear separation between groups at both the transcriptome and proteome levels, indicating molecular alterations induced by 9,10-DiOH treatment (Fig. 3A). Pathway enrichment analysis revealed enrichment of copper-related pathways among the top-ranked pathways, with concordance between transcriptomic and proteomic datasets (Fig. 3B–D). Gene set enrichment analysis further suggested activation of the cellular response to copper ions (Fig. 3E). Several cuproptosis-associated genes, including ferredoxin 1 and solute carrier family 31 member 1, showed positive correlations across both omics layers. To explore a potential interaction, molecular docking analysis was conducted and suggested a possible binding interaction between 9,10-DiOH and ferredoxin 1 (Fig. 3F). Functional validation was subsequently performed using small interfering RNA-mediated knockdown of ferredoxin 1, with the most efficient sequence selected for downstream experiments (Fig. 3G and H). Immunofluorescence analysis demonstrated that 9,10-DiOH increased intracellular copper accumulation, an effect that was attenuated by the copper chelator penicillamine and enhanced by the copper ionophore elesclomol, while ferredoxin 1 knockdown reduced copper accumulation induced by 9,10-DiOH (Fig. 3I). Consistently, quantitative PCR and western blot analyses showed changes in ferredoxin 1, solute carrier family 31 member 1, and lipoylated dihydrolipoamide S-acetyltransferase levels under FDX1 knockdown and copper modulation conditions (Fig. 3J–L). Together, the integrated omics results, molecular docking analysis, FDX1 knockdown, and copper modulation experiments support the involvement of an FDX1-associated copper-dependent/cuproptosis-related regulatory process in the biological effects of 9,10-DiOH. Rather than relying solely on transcriptomic/proteomic correlations, these functional validation experiments further indicate that 9,10-DiOH-mediated intracellular copper accumulation and cuproptosis-associated marker changes are at least partially dependent on FDX1 and copper availability.

### 3.4 9,10-DiOH suppresses osteoclastogenic signaling through FDX1-dependent inhibition of NF-kB and MAPK pathways

**Fig. 4:**
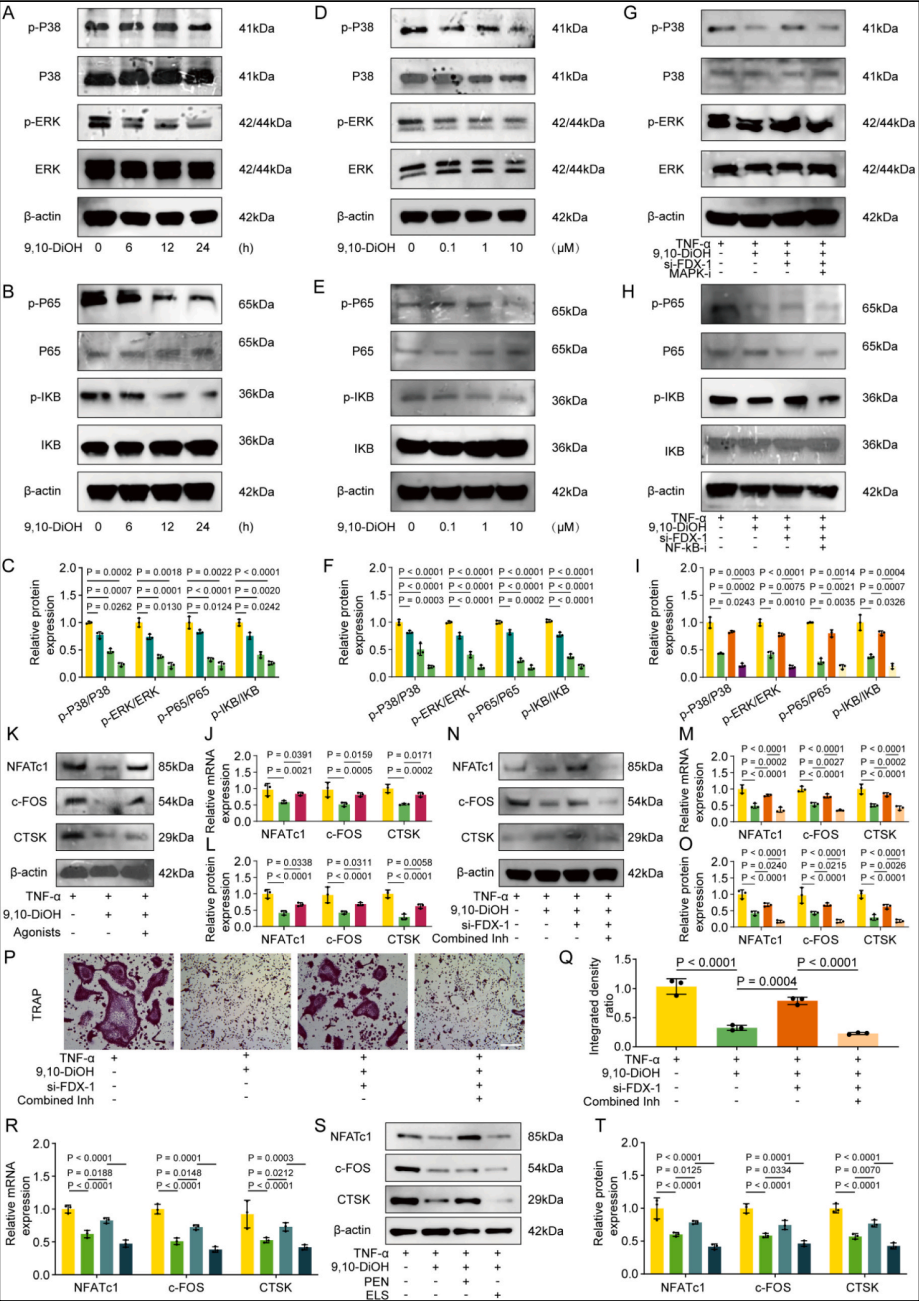
**9,10-DiOH inhibits osteoclastogenic signaling *via* FDX1-dependent suppression of NF-kB and MAPK pathway**s. (**A-C)** Phosphorylation levels and statistical analysis of MAPK and NF-kB pathways at 0, 6, 12, and 24 h post-treatment with 9,10-DiOH. **(D**–**F)** Phosphorylation levels and statistical analysis of MAPK and NF-kB pathways following treatment with 9,10-DiOH at concentrations of 0, 0.1, 1, and 10um. **(G**–**I)** Western blot analysis of MAPK and NF-kB phosphorylation levels. Cells were treated with 9,10-DiOH alone, in combination with FDX1 knockdown, or with the additional application of specific pathway inhibitors. **(J**–**L)** Western blot and quantitative PCR analyses of osteoclast-associated markers nuclear factor of activated T cells 1, c-Fos, and cathepsin K following treatment with pathway agonists. **(M**–**O)** Western blot and quantitative PCR analyses of osteoclast-associated markers nuclear factor of activated T cells 1, c-Fos, and cathepsin K following ferredoxin 1 silencing followed by treatment with pathway agonists. **(P**–**Q)** Representative tartrate-resistant acid phosphatase staining images and corresponding quantitative analysis (scale bar = 15 μm). **(R)** qPCR analysis of NFATc1, c-FOS, and CTSK mRNA expression in osteoclasts treated with 9,10-DiOH, PEN (copper chelator), or ELS (copper ionophore). **(S)** Representative western blot images showing protein expression of NFATc1, c-FOS, and CTSK under the same treatment conditions. **(T)** Quantification of NFATc1, c-FOS, and CTSK protein levels by western blot (n = 3).

To determine whether the anti-inflammatory effects of 9,10-DiOH were mechanistically linked to osteoclastogenic signaling, phosphorylation of key components within the MAPK and NF-kB pathways was examined. Immunofluorescence analysis revealed a marked reduction in phosphorylated p38 and phosphorylated p65 levels following 9,10-DiOH treatment compared with phosphate-buffered saline controls (Supplementary Fig. S3). Western blot results and statistical analysis showed that the phosphorylation levels of the MAPK and NF-kB pathways gradually decreased over time following treatment with 9,10-DiOH at 0 h, 6 h, 12 h, and 24 h (Fig. 4A–C). Furthermore, Western blot results and statistical analysis revealed that the phosphorylation of MAPK and NF-kB pathways decreased gradually in a dose-dependent manner following treatment with 9,10-DiOH at concentrations of 0, 0.1, 1, and 10 μM. (Fig. 4D–F). Importantly, silencing FDX1 significantly abrogated the inhibitory effect of 9,10-DiOH, leading to upregulated phosphorylation of MAPK and NF-kB. However, subsequent treatment with specific inhibitors for these pathways reversed this upregulation in FDX1-deficient cells. Collectively, these results suggest that 9,10-DiOH inhibits MAPK and NF-kB signaling in an FDX1-dependent manner (Fig. 4G–I). Furthermore, downstream osteoclast-related transcription factors and effector molecules, including nuclear factor of activated T cells 1, c-Fos, and cathepsin K, were significantly downregulated at both mRNA and protein levels following 9,10-DiOH treatment, while this downregulation was effectively reversed by the administration of pathway agonists (Fig. 4J–L). Notably, FDX1 knockdown significantly restored the expression of osteoclast-related proteins even in the presence of 9,10-DiOH, whereas subsequent treatment with inhibitors effectively reversed this upregulation. These findings indicate that 9,10-DiOH suppresses osteoclastogenic protein expression by inhibiting MAPK and NF-kB signaling in an FDX1-dependent manner (Fig. 4M and O). Functional validation via TRAP staining further corroborated that 9,10-DiOH inhibits osteoclast formation, a process modulated by the aforementioned mechanisms (Fig. 4P and Q). Collectively, these results indicate that 9,10-DiOH suppresses osteoclastogenic signaling by inhibiting NF-kB and MAPK pathway activation through an FDX1-dependent cuproptosis mechanism. To further determine whether copper-dependent regulation was involved in the anti-osteoclastogenic effect of 9,10-DiOH, PEN and ELS were introduced into the osteoclastogenic system. RT-qPCR analysis showed that 9,10-DiOH significantly reduced the mRNA expression of NFATc1, c-FOS, and CTSK. PEN partially reversed this inhibitory effect, whereas ELS further enhanced the suppression of these osteoclast-related genes (Fig. 4R). Consistently, western blot analysis demonstrated similar changes at the protein level, with PEN restoring and ELS further decreasing NFATc1, c-FOS, and CTSK expression in the presence of 9,10-DiOH (Fig. 4S and T). These findings provide additional evidence that the inhibitory effect of 9,10-DiOH on osteoclastogenic marker expression is closely associated with copper-dependent regulation.

### 3.5 9,10-DiOH attenuates inflammatory responses by limiting FLSs proliferation in a copper-dependent manner

**Fig. 5:**
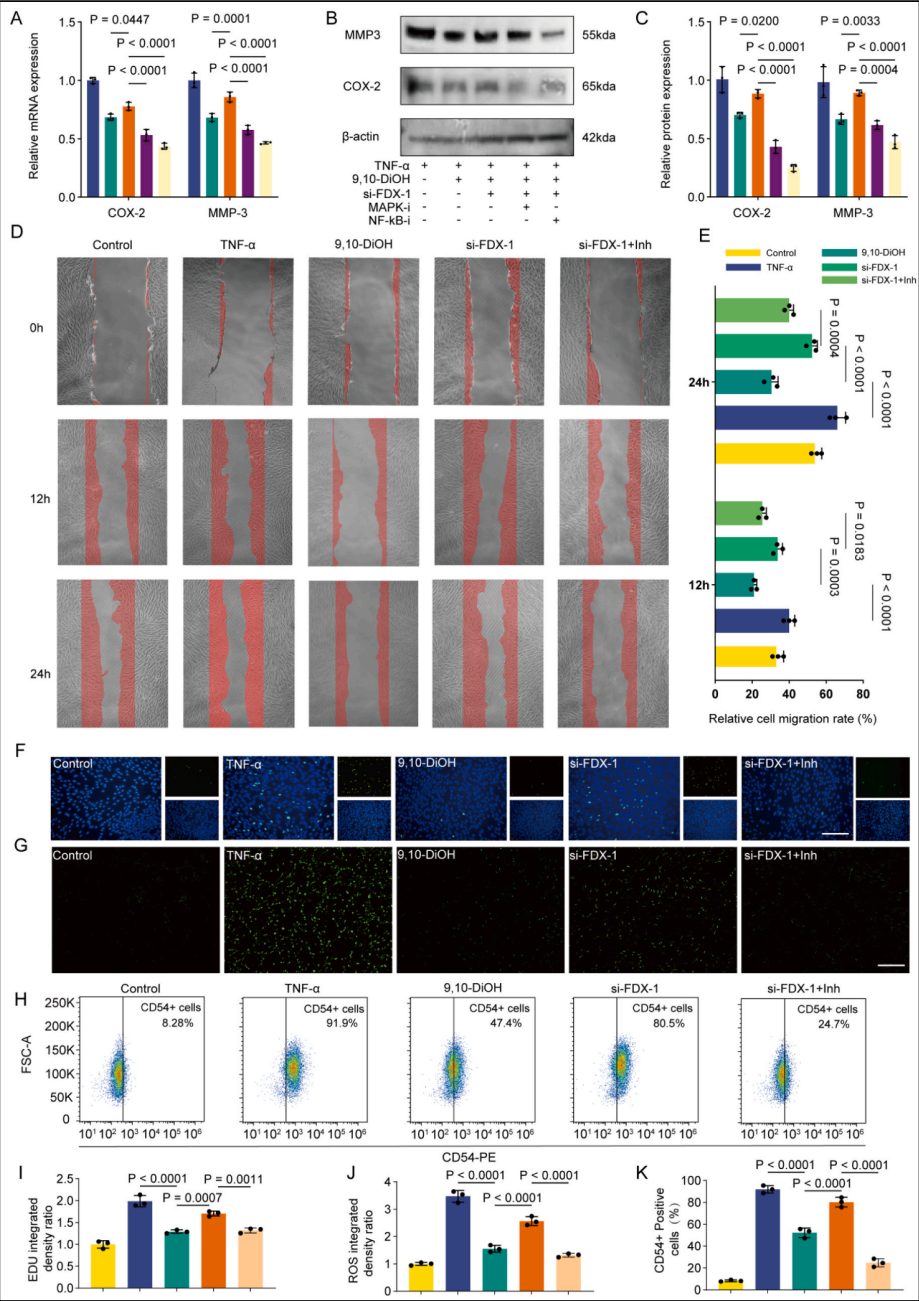
**9,10-DiOH suppresses FLSs proliferation and inflammatory activation in a copper-dependent manner.** (A) Quantitative PCR analysis of cyclooxygenase-2 and matrix metalloproteinase-3 mRNA expression. **(B**–**C)** Western blot analysis of cyclooxygenase-2 and matrix metalloproteinase-3 protein levels with corresponding densitometric quantification. **(D**–**E)** Representative images of scratch wound-healing assays performed on fibroblast-like synoviocytes treated with indicated conditions. **(F)** Representative 5-ethynyl-2′-deoxyuridine incorporation images and quantitative analysis of EdU-positive cells (scale bar = 50 μm). **(G)** Immunofluorescence staining of intracellular reactive oxygen species. **(H)** Flow cytometric analysis of CD54 expression. **(I)** Corresponding quantification of relative cell migration rates at 12 and 24 h. **(J)** Corresponding fluorescence intensity quantification. (K) Statistical summary of CD54-positive cell percentages.

To further investigate the effects of 9,10-DiOH on FLSs behavior, we first performed PCR and WB assays to verify the expression levels of inflammatory mediators before and after treatment, as well as the regulatory relationship between FDX1 knockdown and signaling pathways (Fig. 5A–C). Subsequently, scratch wound-healing and EdU proliferation assays demonstrated that 9,10-DiOH inhibits cell migration and proliferation in an FDX1-dependent manner (Fig. 5D–F). Given the close association between oxidative stress and inflammatory activation, intracellular reactive oxygen species levels were examined and found to be significantly reduced upon 9,10-DiOH exposure, whereas copper chelation or copper ionophore treatment respectively restored or further diminished reactive oxygen species signals (Fig. 5 G). Flow cytometric analysis indicated that 9,10-DiOH significantly reduced cellular inflammation through this signaling pathway. (Fig. 5H). Collectively, these results indicate that 9,10-DiOH suppresses FLSs proliferation and inflammatory activation through an FDX1-dependent, copper-regulated mechanism.

### 3.6 Design and characterization of injectable chitosan-based hydrogels

**Fig. 6:**
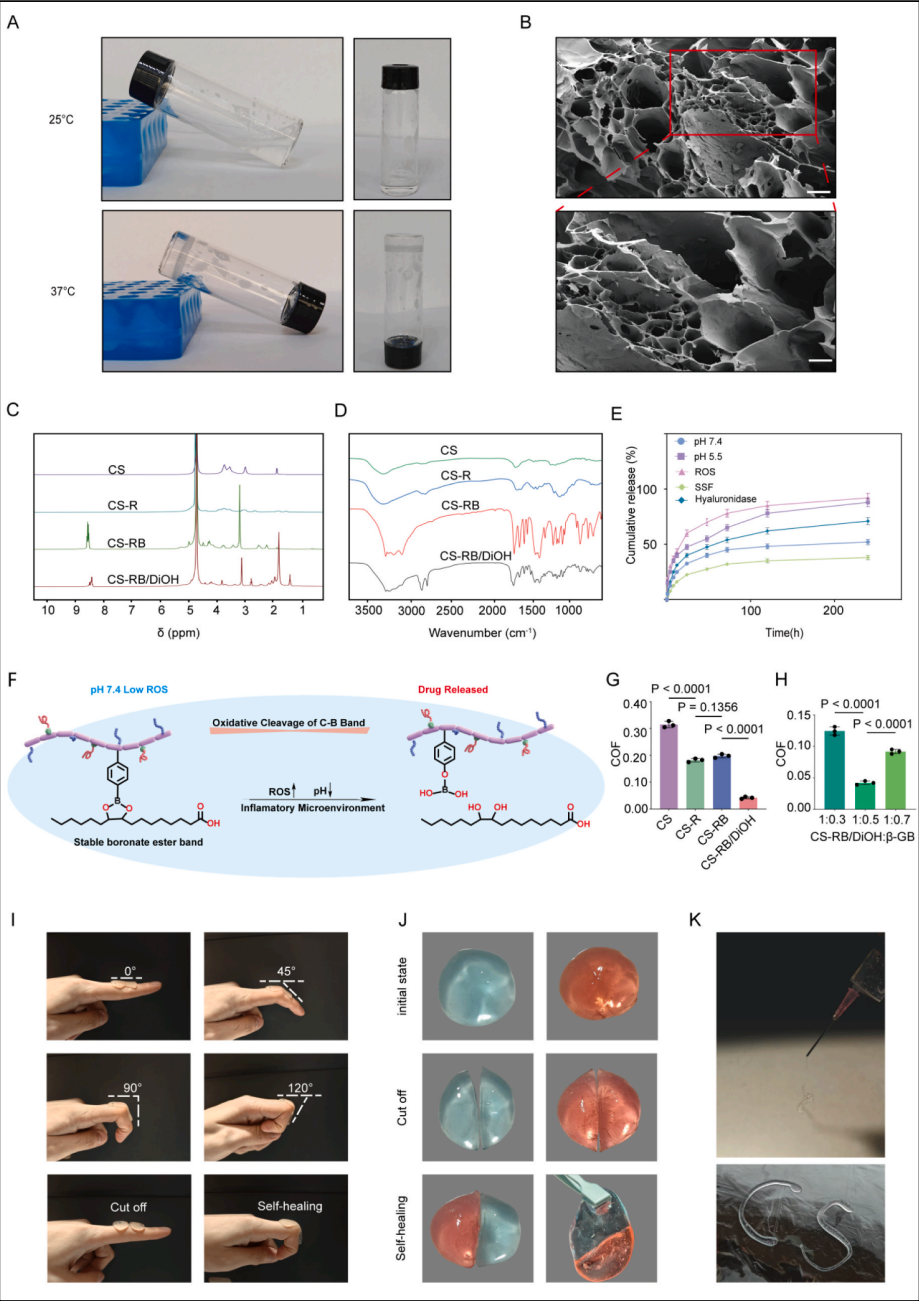
**Synthesis, Characterization, and Stimuli-Responsive Properties of the CS-RB/DiOH Hydrogel**. (**A)** Physical image of CS-RB/DiOH. **(B)** Scanning electron microscopy (SEM) images of the freeze-dried thermosensitive injectable hydrogel at different magnifications, Scale bars: 100 μm (top) and 50 μm (bottom). **(C)**^1^H NMR spectra of CS, CS-R, CS-RB, and CS-RB/DiOH.**(D)** FTIR spectra of CS, CS-R, CS-RB, and CS-RB/DiOH. **(E)***In vitro* multi-stimuli-responsive release profiles of 9,10-DiOH from CS-RB/DiOH hydrogel.Cumulative release (%) was normalized to the total loaded amount (determined as 2.1 μM). The hydrogel exhibited markedly accelerated release under acidic (pH 5.5) and ROS (100 μM H_2_O_2_) conditions mimicking the RA joint microenvironment, reaching therapeutic concentrations of 1.10 μM and 1.40 μM at 48 h, respectively. In simulated synovial fluid (SSF: pH 7.4 PBS containing 2% BSA and 0.3% HA), release was slower due to increased viscosity and protein binding, yet remained significantly responsive to triggers. Enzymatic conditions (hyaluronidase 1500 U mL^−1^) resulted in mild acceleration, confirming controlled long-term delivery over 10 days. **(F)** ROS-triggered oxidative cleavage of boronate ester bonds and disease-responsive drug release mechanism. **(G)** Tribological evaluation showing the steady-state coefficient of friction (COF) of the hydrogels following sequential chemical modifications (CS, CS-R, CS-RB, and CS-RB/DiOH). The physical crosslinking ratio was fixed at the optimal β-GB ratio of 1:0.5. **(H)** COF of the final CS-RB/DiOH hydrogel formulated with varying mass ratios of the thermosensitive crosslinker β-GB (1:0.3, 1:0.5, and 1:0.7).All tribological tests were conducted under a 1 N normal load and 1 Hz reciprocating sliding frequency at 37°C. Data are presented as mean ± SD (n = 3). Statistical significance was determined using one-way ANOVA **(I)**Macroscopic evaluation of the tissue adhesiveness and dynamic flexibility of the hydrogel on a human finger joint during repeated bending at various angles (0°, 45°, 90°, and 120°). **(J)** Visual demonstration of the macroscopic self-healing capacity, showing two distinctly colored hydrogel pieces (blue and red) rapidly fusing into an integrated gel capable of withstanding mechanical stretching after being cut.**(K)**Photographs illustrating the excellent injectability of the hydrogel precursor solution, effortlessly extruding through a fine needle to write the letters “CS”. (For interpretation of the references to color in this figure legend, the reader is referred to the Web version of this article.)

This study details the design and characterization of a thermosensitive, pH/ROS dual-responsive, and actively targeted injectable hydrogel system [16,17]. The hydrogel utilizes chitosan (CS) as the primary polymeric backbone, which is sequentially functionalized with RGD peptides (CS-R) to confer active targeting towards integrin-overexpressing fibroblast-like synoviocytes (FLSs) [18], and phenylboronic acid (CS-RB) to serve as a stimuli-responsive crosslinker. Since RGD-mediated targeting relies on the recognition of integrin receptors expressed on the cell membrane, we further examined the basal expression of integrin β in FLSs. RT-qPCR and western blot analyses confirmed detectable integrin β expression in FLSs, supporting the feasibility of RGD-mediated integrin targeting by the hydrogel system (Supplementary Fig. S4). The therapeutic lipid, 9,10-DiOH, is then conjugated via dynamic covalent boronate ester bonds. Through the addition of β-glycerophosphate (β-GP), this composite material transitions into a localized “drug depot” upon intra-articular injection at physiological temperature (37°C). The localized microenvironment of the rheumatoid arthritis (RA) joint characterized by low pH and elevated reactive oxygen species (ROS) triggers the cleavage of these boronate ester bonds, enabling the precise, disease-responsive release of 9,10-DiOH directly to the pathological site (Fig. 6F).

Regarding material characterization, scanning electron microscopy (SEM) images (Fig. 6B) show the microstructure of the freeze-dried CS-RB/DiOH hydrogel, displaying uniformly distributed pores. This structural integrity and stable porosity are crucial for controlling drug release and ensuring optimal injectability. The ^1^H nuclear magnetic resonance (NMR) spectrum (Fig. 6C) confirms the stepwise chemical modification of the chitosan backbone. Compared to pristine CS, the spectrum of CS-R exhibits a distinct new resonance peak at 3.2 ppm, corresponding to the specific amino acid residues of the RGD peptide. Subsequent modification to CS-RB introduces sharp new aromatic proton signals between 8.5 and 8.8 ppm, verifying the successful grafting of phenylboronic acid moieties. The stable incorporation of 9,10-DiOH within the network is further confirmed by the appearance of prominent aliphatic resonances attributable to its long alkyl chain, specifically the sharp peaks at 1.3 ppm and 1.8 ppm. Fourier-transform infrared spectroscopy (FTIR) analysis (Fig. 6D) corroborates these structural changes. The successive modifications exhibit progressively strengthened amide-related absorption bands. Notably, CS-RB displays highly intense and sharp characteristic peaks in the 1300-1700 cm^−1^ region, indicating the successful crosslinking of CPBA. Furthermore, the CS-RB/DiOH spectrum displays remarkably distinct aliphatic C-H stretching doublet bands at 2920 and 2850 cm^−1^, alongside characteristic changes in the B-O/C-O stretching region (1350-1100 cm^−1^), explicitly confirming the formation of dynamic boronate ester bonds between the hydrogel and the drug.

Drug release behavior analysis (Fig. 6E) reveals that the hydrogel exhibits robust responsiveness under disease-mimicking (pH 5.5) and ROS environments compared to physiological conditions (pH 7.4). At a lower pH, typical of an inflammatory microenvironment, the phenylboronic acid groups undergo protonation, destabilizing the boronate ester bonds and promoting rapid drug release. Under physiological pH (7.4), these groups remain unprotonated, effectively retaining the encapsulated drug. Furthermore, the ROS responsiveness stems from the oxidative cleavage of the carbon-boron bonds within the hydrogel matrix, providing a secondary trigger for drug release under oxidative stress [19]. This dual-responsive mechanism ensures precise spatial and temporal control over drug delivery.

Physical observations were initially conducted to evaluate the macroscopic properties of the hydrogel (Fig. 6A). To validate its thermosensitivity, a dye-labeled formulation was injected as a flowable liquid at room temperature into a 37°C water bath.As demonstrated in Video 1, the solution rapidly transitioned into a solid gel state, verifying its thermoresponsive behavior. This localized gelation is essential for injectable therapeutics, ensuring that the formulation acts as a stable, long-term intra-articular depot. Furthermore, macroscopic evaluations visually validated the mechanical and practical advantages of the hydrogel. As shown in (Fig. 6K), the precursor solution exhibited excellent injectability, easily extruding through a fine needle to continuously write the letters “CS” without clogging. Importantly, the formed hydrogel demonstrated robust tissue adhesiveness and dynamic flexibility. When applied to a human finger joint, it maintained conformal contact and structural integrity without detachment during repeated bending and straightening (Fig. 6I), which is highly desirable for withstanding continuous mechanical movements within an active RA joint. Finally, the remarkable self-healing capacity observed in the rheological strain-recovery tests was macroscopically corroborated: when two distinctly colored hydrogel pieces (blue and red) were cut in half and placed in physical contact, they rapidly fused into a single, integrated hybrid gel capable of withstanding mechanical stretching (Fig. 6J). This macroscopic healing is attributed to the reversible reformation of the dynamic boronate ester bonds within the network [20].

Supplementary data related to this article can be found online at https://doi.org/10.1016/j.mtbio.2026.103502

The following are the Supplementary data related to this article:

Multimedia component 2

Rheological evaluations further verified the thermosensitivity, injectability, and self-healing properties of the system. Temperature-ramp tests evaluating the storage modulus (G′) and loss modulus (G″) of hydrogels at various formulation ratios (1:0.3, 1:0.5, 1:0.7) demonstrated a rapid increase in G’ upon heating, indicating excellent thermal responsiveness and a sharp sol-gel transition (Supplementary Fig. 5G–J). Additionally, strain sweep experiments (Supplementary Fig. 5A–C) and time-strain recovery tests (Supplementary Fig. 5D–F) established that the hydrogel rapidly recovers its initial storage modulus following cyclical high-strain deformation.

Beyond the physical crosslinking ratio, the sequential chemical functionalization profoundly enhanced the biomimetic lubrication (Fig. 6G). At the optimal 1:0.5 ratio, the COF decreased from 0.315 ± 0.011 for the pristine CS hydrogel to 0.182 ± 0.006 for CS-R, owing to the hydration lubrication provided by the highly polar RGD peptides trapping a water layer at the interface [21]. Notably, the final CS-RB/DiOH hydrogel achieved an ultralow COF of 0.042 ± 0.004. This dramatic reduction is attributed to the 18-carbon hydrophobic alkyl chains of 9,10-DiOH, which self-assemble at the sliding interface to form a robust boundary lubricating film, structurally mimicking the natural lipid layer of articular cartilage [22].

Crucially, this crosslinking density directly dictates the tribological performance of the hydrogels (Fig. 6H). Varying the β-GP ratio revealed a U-shaped coefficient of friction (COF) trend. The optimal 1:0.5 ratio exhibited the lowest friction, striking a perfect balance between mechanical robustness and spatial freedom for lipid mobility. Conversely, lower (1:0.3) or higher (1:0.7) ratios led to increased friction (0.124 and 0.091, respectively) due to either insufficient mechanical support under load or restricted lipid chain movement caused by an overly dense polymeric network.

In summary, this CS-RB/DiOH hydrogel functions as a sophisticated dual-targeting drug delivery system. It combines macro-level intra-articular retention via thermosensitive gelation with micro-level cellular targeting via RGD peptides. Driven by pH and ROS-responsive drug release, alongside outstanding rheological, self-healing, and biomimetic lubricating properties, it represents a highly precise and robust platform for RA therapy.

### 3.7 Hydrogel encapsulation improves biosafety and enhances the anti-inflammatory efficacy of 9,10-DiOH

**Fig. 7:**
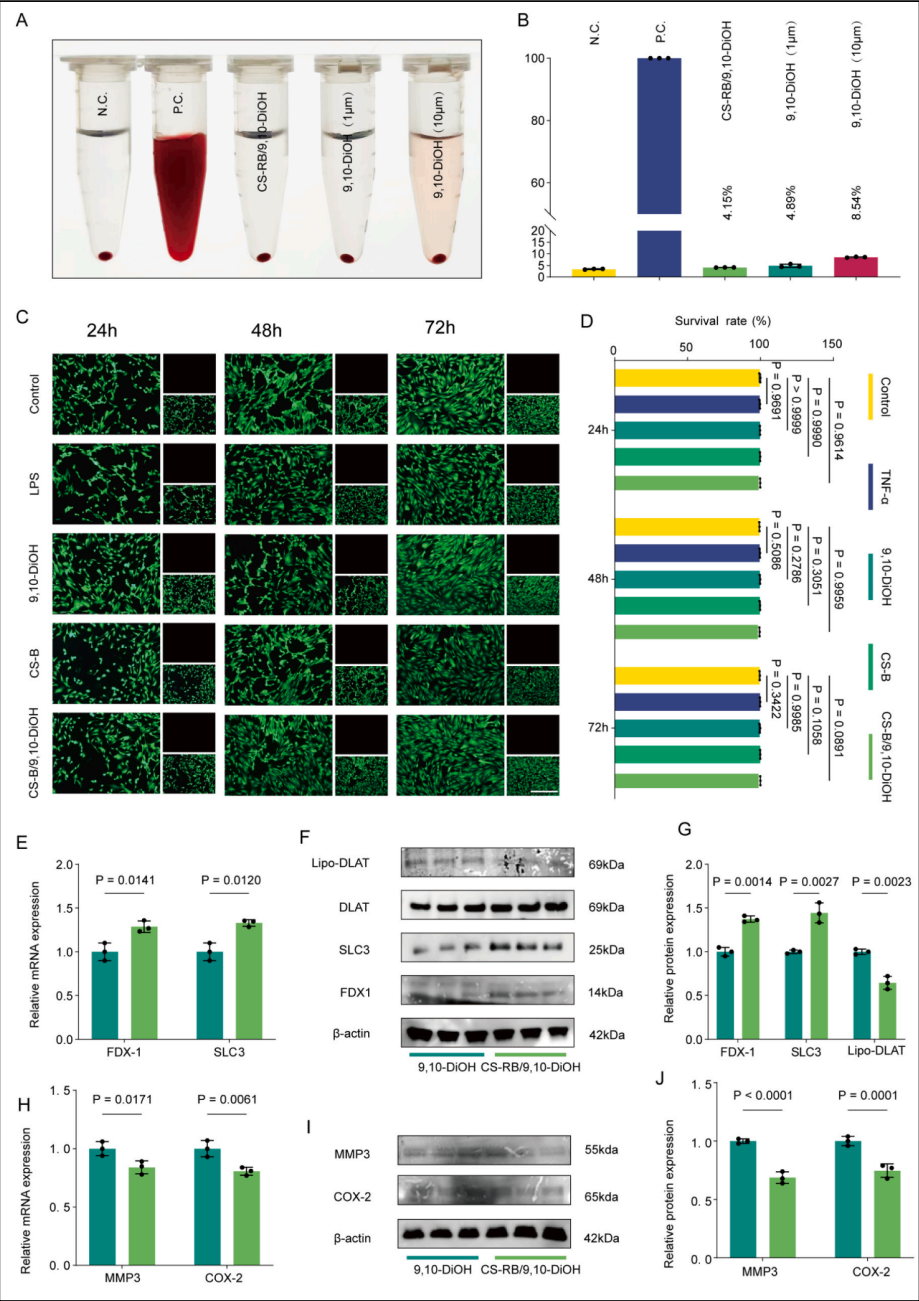
**Biosafety evaluation and enhanced anti-inflammatory activity of the CS-RB/DiOH hydrogel syst**em. (**A-B)** Representative images of hemolysis assays performed with negative control (normal saline), positive control (distilled water), CS-B/DiOH hydrogel, and free 9,10-DiOH at 1 μM and 10 μM with corresponding quantitative analysis of hemolysis percentage. **(C**–**D)** Representative live/dead staining images of cells cultured with indicated treatments at 24, 48, and 72 h, and corresponding cell survival rate quantification (scale bar = 50 μm). **(E)** Quantitative PCR analysis of FDX1 and SLC3A1 expression. **(F**–**G)** Western blot analysis of FDX1, SLC3A1, and Lipo-DLAT protein levels, with densitometric quantification. **(H)** Quantitative PCR analysis of inflammatory markers MMP3 and COX-2. (I-J) Western blot analysis of MMP3 and COX-2 protein expression, with corresponding quantitative analysis.

To evaluate the biosafety and functional performance of the hydrogel-based 9,10-DiOH delivery system, hemolysis assays were first conducted. Compared with the negative control (normal saline) and positive control (distilled water), the CS-B/DiOH and free 9,10-DiOH at different concentrations exhibited negligible hemolytic activity, confirming favorable blood compatibility (Fig. 7A and B). Live/dead staining performed at 24, 48, and 72 h further demonstrated that the hydrogel formulation did not induce obvious cytotoxicity, with cell viability comparable to control conditions (Fig. 7C and D). At the molecular level, quantitative PCR and western blot analyses revealed that encapsulation of 9,10-DiOH within the hydrogel significantly enhanced the expression of cuproptosis-related proteins FDX1 and SLC3A1 compared with free drug administration (Fig. 7E–G). Meanwhile, expression of inflammatory mediators matrix metalloproteinase-3 and cyclooxygenase-2 was markedly reduced at both the transcriptional and protein levels following hydrogel treatment, exceeding the anti-inflammatory efficacy observed with free 9,10-DiOH alone (Fig. 7H–J). Collectively, these results indicate that the hydrogel-based delivery system not only exhibits excellent biocompatibility but also amplifies the cuproptosis-associated and anti-inflammatory effects of 9,10-DiOH.

### 3.8 The hydrogel composite enhances therapeutic efficacy in a murine model of rheumatoid arthritis

**Fig. 8:**
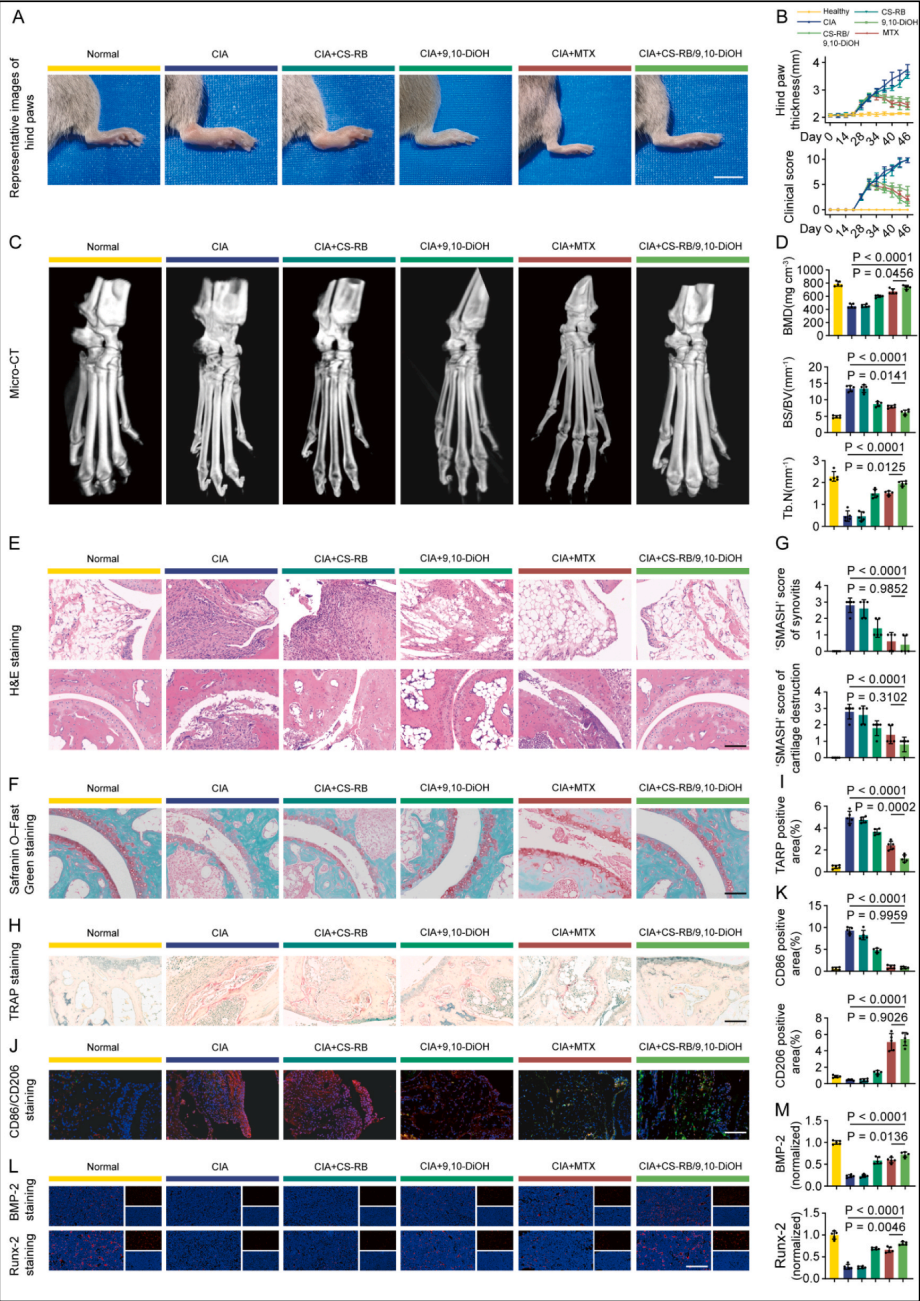
**The hydrogel composite alleviates joint inflammation and bone destruction in CIA mice**. (**A)** Representative gross images of hind paws from different treatment groups. **(B)** Quantification of hind paw thickness and clinical arthritis scores. **(C)** Representative Micro-CT images of hind limb joints. **(D)** Quantitative Micro-CT analysis of bone parameters, including BMD, bone surface to bone volume ratio (BS/BV), and trabecular number (Tb.N). **(E)** H&E staining of joint sections. **(F)** Safranin O-Fast Green staining of cartilage. **(G)** Semi-quantitative scoring of synovial inflammation and cartilage destruction. **(H)** TRAP staining of osteoclasts. **(I)** Quantification of TRAP-positive cells. **(J)** Immunofluorescence staining of macrophage markers CD86 and CD206. **(K)** Quantification of CD86^−^and CD206-positive cells. **(L)** Immunofluorescence staining of osteogenic markers BMP-2 and Runx-2. **(M)** Quantitative analysis of BMP-2 and Runx-2 expression. (For interpretation of the references to color in this figure legend, the reader is referred to the Web version of this article.)

To further evaluate the therapeutic potential of the hydrogel composite *in vivo*, a CIA mouse model was established, and methotrexate (MTX) was included as a positive control. Representative gross images showed that both MTX and hydrogel composite treatment reduced hind paw swelling compared with the CIA group and single-agent treatment groups (Fig. 8A). Quantitative analysis further confirmed significant reductions in paw thickness and clinical arthritis scores after treatment, with the hydrogel composite exhibiting a stronger therapeutic effect than MTX (Fig. 8B). Micro-CT analysis demonstrated that the hydrogel composite effectively alleviated joint bone destruction, as evidenced by improved preservation of bone architecture (Fig. 8C). Notably, quantitative bone parameters, including BMD, BS/BV, and Tb.N, were significantly improved in the hydrogel composite-treated group, and these improvements were more pronounced than those observed in the MTX group (Fig. 8D). Histopathological examination using H&E staining revealed attenuated synovial hyperplasia and reduced inflammatory cell infiltration after treatment (Fig. 8E). Safranin O-Fast Green staining further indicated preservation of cartilage structure and reduced proteoglycan loss compared with arthritic controls (Fig. 8F), which was supported by decreased synovitis and cartilage destruction scores. No significant difference in these histological scores was observed between the hydrogel composite and MTX groups (Fig. 8G). TRAP staining showed a marked reduction in osteoclast numbers following treatment, and quantitative analysis revealed that the hydrogel composite suppressed osteoclast formation more effectively than MTX (Fig. 8H and I). In addition, immunofluorescence analysis revealed a decreased proportion of CD86-positive cells and an increased proportion of CD206-positive cells within joint tissues, indicating a shift toward an anti-inflammatory macrophage phenotype. The macrophage polarization-related therapeutic effect of the hydrogel composite was comparable to that of MTX (Fig. 8J and K). Finally, immunofluorescence staining demonstrated elevated expression of the osteogenic markers BMP-2 and Runx-2, with the hydrogel composite showing a stronger enhancement than MTX, suggesting superior restoration of osteogenic activity in arthritic joints (Fig. 8L and M).

To further evaluate the systemic biosafety of CS-RB/9,10-DiOH, serum biochemical indicators and histological changes in major organs were examined. Serum biochemical analysis showed no significant differences in liver function-related indicators, including ALB, TBIL, ALT, AST, and γ-GT, between the CIA and CS-RB/9,10-DiOH-treated groups. In addition, lipid metabolism, glucose metabolism, renal function, and electrolyte-related indicators, including TG, CHOL, GLU, BUN, CRE, Ca, and P, remained within comparable ranges after CS-RB/9,10-DiOH treatment (Supplementary Fig. S6). Furthermore, H&E staining of the heart, liver, spleen, lung, and kidney showed no obvious pathological abnormalities, inflammatory infiltration, tissue necrosis, or structural damage in mice treated with CS-RB, free 9,10-DiOH, or CS-RB/9,10-DiOH compared with the normal group (Supplementary Fig. S7). These results suggest that CS-RB/9,10-DiOH exhibited favorable systemic biosafety during *in vivo* administration.

## 4 Discussion

RA is a chronic systemic autoimmune disorder characterized by persistent synovial inflammation, aggressive proliferation of FLSs, excessive osteoclast activation, and progressive joint destruction [23]. The pathological interplay between synovial hyperplasia, inflammatory signaling, and bone erosion forms a self-amplifying vicious cycle that ultimately leads to irreversible joint damage. Although current therapeutic strategies-including disease-modifying antirheumatic drugs and biologics-have significantly improved clinical outcomes, their long-term efficacy is often compromised by immunosuppression-related adverse effects, high costs, and the development of drug resistance [[24], [25], [26]]. Consequently, there is a growing interest in identifying novel therapeutic targets that modulate disease progression through noncanonical mechanisms, particularly those linked to cellular metabolism and stress responses [27,28].

Emerging epidemiological and experimental evidence suggests that lipid metabolism plays a critical role in autoimmune diseases [29]. Notably, populations with long-term dietary intake of fatty acids exhibit a reduced incidence of autoimmune disorders, including RA. However, the precise bioactive components and molecular mechanisms underlying these protective effects remain poorly understood. In this study, through systematic component analysis of lipid-rich formulations, we identified 9,10-DiOH as a key functional lipid mediator with pronounced anti-inflammatory and anti-osteoclastic effects. Our findings provide evidence that 9,10-DiOH alleviates RA progression by engaging FDX1-associated copper-dependent regulation (cuproptosis), thereby reshaping inflammatory signaling and bone remodeling processes.

Metabolic dysregulation is increasingly recognized as a hallmark of RA pathogenesis. Clinical studies have reported abnormal alterations in systemic metabolic parameters in RA patients, including elevated levels of circulating copper ions. Copper is an essential trace element involved in mitochondrial respiration and redox balance; however, excessive intracellular copper accumulation can induce cytotoxicity. This dual nature of copper prompted us to investigate whether copper metabolism is mechanistically linked to RA-associated metabolic abnormalities. Our metabolomic analyses revealed a pronounced upregulation of the tricarboxylic acid (TCA) cycle following 9,10-DiOH treatment, indicating enhanced mitochondrial oxidative metabolism. These findings suggest that RA progression is closely associated with altered energy metabolism and that restoration of mitochondrial function may represent a therapeutic entry point.

Consistent with these observations, mitochondrial functional assays demonstrated that mitochondrial functional assays demonstrated that 9,10-DiOH reduced mitochondrial membrane potential and ATP production, supporting mitochondrial functional impairment rather than merely metabolic remodeling. Given that cuproptosis is a recently described form of regulated cell death driven by copper-dependent aggregation of lipoylated mitochondrial enzymes in the TCA cycle, these results support mitochondrial metabolic reprogramming as a central mechanism underlying the effects of 9,10-DiOH. Importantly, our integrated transcriptomic and proteomic analyses further substantiated this hypothesis by revealing significant enrichment of copper ion-related pathways and metal detoxification processes following drug treatment.

Among the identified pathways, cuproptosis-associated regulators, including FDX1, SLC31A1, and lipoic acid metabolism-related proteins, were altered after 9,10-DiOH treatment. FDX1 has been recognized as an upstream regulator of cuproptosis by modulating copper redox cycling and mitochondrial lipoylated protein accumulation. In our study, transcriptomic and proteomic analyses showed increased FDX1 expression following 9,10-DiOH treatment, while molecular docking suggested a potential interaction between 9,10-DiOH and FDX1. Functional validation using siRNA-mediated FDX1 knockdown further indicated that FDX1 contributes to the effects of 9,10-DiOH, as FDX1 silencing attenuated intracellular copper accumulation and partially weakened the downstream biological responses induced by 9,10-DiOH.

Inflammatory signaling pathways, particularly NF-κB and MAPK, play pivotal roles in RA pathogenesis by driving cytokine production, FLS activation, and osteoclast differentiation. Our data showed that 9,10-DiOH reduced the phosphorylation of key components within both NF-κB and MAPK pathways in a time- and dose-dependent manner. Copper modulation experiments using penicillamine and elesclomol further supported the involvement of copper-dependent regulation in this process. However, the current data do not fully determine whether NF-κB/MAPK inhibition is a direct primary consequence of copper-dependent mitochondrial stress or a secondary downstream effect of altered cellular metabolism. Therefore, we interpret these findings as evidence that FDX1-associated copper-dependent/cuproptosis-related regulation is linked to reduced NF-κB/MAPK activation. Further studies are needed to define the precise molecular intermediates connecting copper-dependent mitochondrial stress with inflammatory signal transduction in RA.

Excessive osteoclastogenesis is a major driver of bone erosion in RA. NF-kB and MAPK signaling pathways are essential for receptor activator of nuclear factor kB ligand (RANKL)-induced osteoclast differentiation [30]. In line with this, we observed that 9,10-DiOH markedly downregulated the expression of osteoclast-related transcription factors and effector proteins, including NFATc1, c-FOS, and CTSK. Genetic and pharmacological disruption of cuproptosis signaling restored osteoclast marker expression and bone-resorptive activity, further confirming that cuproptosis activation acts upstream of osteoclast inhibition. These results indicate that 9,10-DiOH suppresses osteoclastogenesis by attenuating NF-kB/MAPK signaling through an FDX1-dependent cuproptosis mechanism.

Beyond its effects on osteoclasts, 9,10-DiOH also exerted profound regulatory effects on FLSs behavior. Aberrant proliferation, migration, and inflammatory activation of FLSs are central to synovial hyperplasia and pannus formation [31]. Our wound-healing and EdU assays demonstrated that 9,10-DiOH significantly inhibited FLSs migration and proliferation. Concurrently, intracellular ROS levels and inflammatory markers such as CD54, COX-2, and MMP3 were markedly reduced. Notably, these effects were reversed by FDX1 knockdown or copper chelation, underscoring the central role of cuproptosis-mediated metabolic stress in controlling synovial inflammation.

Despite its promising therapeutic potential, the clinical application of small-molecule lipid mediators such as 9,10-DiOH is often limited by poor stability, rapid clearance, and lack of tissue specificity. To address these challenges, we developed a hydrogel-based delivery system capable of sustained release and localized retention within inflamed synovial tissue. *In vitro* biosafety assessments confirmed the excellent hemocompatibility and cytocompatibility of the hydrogel composite. Importantly, the hydrogel-based formulation significantly enhanced the activation of cuproptosis-related proteins while further suppressing inflammatory mediators compared with free drug administration.

Beyond acting merely as a sustained drug reservoir, the rational design of the CS-RB/DiOH hydrogel addresses critical biomechanical and materials-level challenges inherent to RA pathology. A major complication of RA is the accelerated deterioration of articular cartilage, which leads to pathologically high friction and severe mechanical wear within the joint cavity. From a tribological perspective, the sequential chemical functionalization of our hydrogel transforms the macroscopic frictional behavior of the joint interface. The integration of highly polar RGD peptides facilitates hydration lubrication by trapping a water layer, while the strategic tethering of 9,10-DiOH introduces 18-carbon hydrophobic alkyl chains that self-assemble into a resilient boundary lubricating film. This biomimetic architecture significantly reduces the coefficient of friction (COF) to a low COF (∼0.042), effectively mimicking the natural lipid boundary layer of healthy articular cartilage. Furthermore, the dynamic covalent nature of the boronate ester bonds endows the polymeric network with autonomous self-healing capabilities and shear-thinning injectability. This allows the hydrogel to structurally conform to the dynamic geometry of the joint and withstand the relentless mechanical fatigue associated with continuous physical movement. Thus, the synergistic integration of active targeting, intelligent drug release, and favorable biomechanical lubrication provides a comprehensive materials-science solution that mechanically and biologically mitigates structural joint deterioration.

The tribological performance of the hydrogel should be interpreted in the context of RA-associated joint degeneration rather than as an isolated material property. In RA, persistent synovitis and cartilage matrix degradation disrupt the native lubricating boundary layer, thereby increasing local frictional stress and accelerating cartilage wear. Therefore, the reduced coefficient of friction observed for CS-RB/DiOH may help alleviate mechanical irritation at the diseased joint interface and complement the biological effects of 9,10-DiOH on inflammation, osteoclast activity, and bone remodeling. This combined biological and mechanical protection provides a rationale for integrating drug delivery with joint lubrication in RA treatment.

The *in vivo* therapeutic efficacy of the hydrogel composite was validated in RA mouse models. Compared with free 9,10-DiOH treatment, the hydrogel formulation markedly reduced paw swelling and clinical scores, attenuated synovial inflammation, and preserved joint architecture. Micro-CT analysis demonstrated significant improvements in bone mineral density and trabecular parameters, while histological staining revealed reduced cartilage destruction and osteoclast activity. Furthermore, macrophage polarization analysis indicated a shift from a pro-inflammatory CD86^+^ phenotype toward an anti-inflammatory CD206^+^ phenotype, accompanied by enhanced expression of osteogenic markers such as BMP-2 and Runx-2. These findings suggest that the hydrogel-based system not only suppresses inflammation and bone resorption but also promotes bone regeneration, contributing to joint homeostasis restoration.

In addition to therapeutic efficacy, the biosafety of intra-articular hydrogel delivery is an important consideration for RA treatment, particularly because repeated local administration may potentially induce synovial irritation, cartilage toxicity, systemic inflammatory responses, or organ toxicity. In the present study, CS-RB/9,10-DiOH showed favorable short-term biocompatibility in vitro, as supported by hemolysis and Live/Dead assays. In vivo, repeated intra-articular administration did not cause obvious systemic biochemical abnormalities, as liver function-, renal function-, glucose/lipid metabolism-, and electrolyte-related serum indicators remained comparable after treatment. H&E staining of the heart, liver, spleen, lung, and kidney further revealed no apparent inflammatory infiltration, tissue necrosis, or structural damage after administration of CS-RB, free 9,10-DiOH, or CS-RB/9,10-DiOH. Serum inflammatory cytokine analysis also indicated that the hydrogel formulation did not induce obvious systemic inflammatory responses. Together with local joint histological evaluation, these findings support the preliminary biosafety of CS-RB/9,10-DiOH for intra-articular delivery.

Nevertheless, the current biosafety and translational evaluation remains preliminary. Although intra-articular injection enables localized retention and the present repeated-injection regimen provides initial evidence of tolerability, the long-term residence, biodegradation behavior, degradation-product safety, potential immune responses, and repeated-injection-related risks of CS-RB/DiOH require further systematic evaluation in larger animals and over longer observation periods. In addition, optimization of formulation stability, sterilization, storage, and scalable manufacturing will be necessary for future translational development.

In summary, this study identifies 9,10-DiOH as a previously unrecognized lipid-derived modulator of RA progression and establishes cuproptosis as a critical regulatory mechanism linking metabolic stress to inflammatory signaling and bone remodeling. By activating the FDX1-dependent cuproptosis pathway, 9,10-DiOH suppresses NF-kB/MAPK signaling, inhibits osteoclastogenesis, restrains synovial hyperplasia, and alleviates joint inflammation. The hydrogel-based delivery strategy further amplifies these therapeutic effects, offering a targeted and sustained treatment approach. Collectively, our findings provide a novel metabolism-based therapeutic paradigm for RA and highlight cuproptosis as a promising target for future autoimmune disease interventions.

## 5 Conclusion

In conclusion, this study identifies 9,10-DiOH as a shared lipid-derived bioactive component with therapeutic potential against RA progression. Mechanistically, 9,10-DiOH engages an FDX1-associated copper-dependent/cuproptosis-related regulatory process, which is accompanied by intracellular copper accumulation, mitochondrial functional impairment, and modulation of cuproptosis-associated markers. These metabolic changes are linked to suppression of NF-κB/MAPK signaling, thereby reducing inflammatory activation of FLSs and inhibiting osteoclastogenic marker expression and osteoclast formation. To improve local delivery and therapeutic durability, 9,10-DiOH was incorporated into an RGD-modified, pH/ROS-responsive, thermosensitive chitosan-based hydrogel. This system integrates targeted delivery, disease-responsive release, and joint-lubricating properties, resulting in reduced synovial inflammation, attenuated bone erosion, and improved joint protection in CIA mice. Overall, this work provides an integrated lipid metabolism- and biomaterial-based strategy for RA intervention while highlighting FDX1-associated copper-dependent regulation as a potential therapeutic axis.

## Ethics approval and consent to participate

All animal procedures were approved by the Institutional Animal Care and Use Committee (IACUC) of Shanghai Jiao Tong University (Protocol No. A2025578)

## Availability of data and materials

All data from this study are available from the corresponding author upon reasonable request.

## Consent for publication

All authors consent to the publication of this manuscript and confirm that all data presented are original and do not include any identifiable information of individuals.

## Funding

This work was supported by the 10.13039/501100001809National Natural Science Foundation of China (No. 82274465).

## CRediT authorship contribution statement

**Yuntao Li:** Conceptualization, Data curation, Formal analysis, Investigation, Methodology, Project administration. **Hongji Liu:** Investigation, Methodology, Resources. **Fangzhou Xie:** Data curation, Funding acquisition. **Hanwen Chang:** Data curation, Methodology. **Yiwei Zhang:** Software, Supervision. **Yuxin Zhang:** Software, Visualization. **Yu Fu:** Investigation, Methodology. **Wenjun Tang:** Software, Supervision, Visualization. **Zengguang Wang:** Resources, Software, Visualization. **Hairong Tao:** Investigation, Methodology, Validation, Writing – original draft, Writing – review & editing. **Xiuping Cheng:** Writing – original draft, Writing – review & editing.

## Declaration of competing interest

The authors declare no potential conflicts of interest with respect to the research, authorship, or publication of this article.

## Data Availability

Data will be made available on request.

## Abbreviations

RA: Rheumatoid arthritis; 9,10-DiOH: 9,10-dihydroxyoctadecanoic acid
CIA: Collagen-induced arthritis
CS: chitosan
FDX1: ferredoxin 1
TRAP: tartrate-resistant acid phosphatase
FLSs: fibroblast-like synoviocytes
NF-kB: nuclear factor kappa B
MAPK: mitogen-activated protein kinase signaling
DAPI: 4′,6-diamidino-2-phenylindole
BMD: bone mineral density
BS/BV: bone surface to bone volume ratio
Tb.N: trabecular number
FITC: Fluorescein isothiocyanate
IHC: Immunohistochemistry
PCA: Principal component analysis
GSEA: Gene set enrichment analysis
CCK-8: Cell counting kit-8
