# Supplementary material for: An intelligent tribological hydrogel delivering 9,10-DiOH activates cuproptosis for comprehensive rheumatoid arthritis intervention
Source: Mater Today Bio. 2026 Jul 29;40:103502. doi: 10.1016/j.mtbio.2026.103502 (PMC13453499; doi:10.1016/j.mtbio.2026.103502)
Supplement: Supplementary file 1 [file mmc1.doc]

**Supplementary Information**

**Title:** **An intelligent tribological hydrogel delivering 9,10-DiOH activates cuproptosis for comprehensive rheumatoid arthritis intervention**

**Supplementary Figures:**

**Fig S1** **Chemical profiling and annotation evidence for 9,10-dihydroxystearic acid in Linwa oil and peach kernel. (A)** Chemical classification of Linwa oil lipid formulation based on untargeted LC-MS/MS analysis. **(B)** Chemical classification of peach kernel lipid formulation based on untargeted LC-MS/MS analysis. (C) Comparative analysis of shared fatty-acyl components detected in both lipid preparations. **(D)** Pathway enrichment analysis of the identified shared bioactive lipid components. **(E)** EIC of the feature assigned to 9,10-dihydroxystearic acid in Linwa oil, detected at RT 30.53 min with m/z 315.2536 in negative ion mode. No MS/MS spectrum was acquired for this feature. **(F)** EIC and MS/MS mirror-spectrum comparison of the feature assigned to 9,10-dihydroxystearic acid in peach kernel, detected at RT 30.53 min with m/z 315.2538 in negative ion mode.

**Fig S2. Effects of 9,10-DiOH on mitochondrial markers in TNF-α or LPS-stimulated cells.** (A–B) Immunofluorescence staining for COX IV (red, mitochondrial inner membrane marker) and TOMM20 (red, mitochondrial outer membrane marker) with DAPI nuclear counterstain (blue). Cells were treated with TNF-α or LPS in the presence or absence of 9,10-DiOH. Quantification of integrated fluorescence intensity is shown in the adjacent bar graphs (scale bar = 50 µm).

**Fig S3 Immunofluorescence characterization of NF-kB and MAPK pathway activation under 9,10-DiOH treatment.** **(A-B)** Immunofluorescence analysis of phosphorylated p65 and phosphorylated p38 in cells treated with phosphate-buffered saline or 9,10-DiOH (scale bar = 50 µm).

**Figure S4 Basal and TNF-α-induced expression profiles of Integrin β1 in fibroblast-like synoviocytes (FLS).** **(A)** Relative mRNA expression level of *ITGB1* in FLS treated with or without TNF-α (10 ng/mL) for 24 h. **(B)** Representative Western blot bands showing the protein levels of Integrin β1and internal control β-actin. **(C)** Quantitative analysis of relative Integrin β1 protein expression normalized against β-actin intensity.

**Fig S5 Rheological properties of thermosensitive injectable hydrogels with different concentrations.** **(A-C)** Strain sweeps at 25 °C and 1 Hz showing storage modulus G' and loss modulus G''for **(A)** 1:0.3, **(B)** 1:0.5, and **(C)** 1:0.7 hydrogels. **(D-F)** Continuous step-strain time sweeps at 25 °C and 1 Hz demonstrating thixotropic and self-healing behavior (alternating between low strain 1% and high strain 500%) for **(D)** 1:0.3, **(E)** 1:0.5 and **(F)** 1:0.7 hydrogels. Rapid recovery of G’ after high strain indicates excellent injectability and self-healing properties. **(G)** 1:0.3 hydrogel, showing storage modulus G’ and loss modulus G’’. **(H)** 1:0.5 hydrogel. **(I)** 1:0.7 hydrogel. Higher concentration results in lower gelation temperature and higher final modulus.

**Figure S6** **Serum biochemical evaluation of CIA mice after CS-RB/9,10-DiOH treatment. (A)** Serum levels of liver function-related biochemical indicators, including albumin (ALB), total bilirubin (TBIL), alanine aminotransferase (ALT), and aspartate aminotransferase (AST). **(B)** Serum levels of γ-glutamyl transpeptidase (γ-GT), triglyceride (TG), total cholesterol (CHOL), and glucose (GLU). **(C)** Serum levels of renal function- and electrolyte metabolism-related indicators, including blood urea nitrogen (BUN), creatinine (CRE), calcium (Ca), and phosphorus (P). No significant differences were observed between the CIA group and the CS-RB/9,10-DiOH-treated group, indicating that CS-RB/9,10-DiOH treatment did not induce obvious systemic biochemical toxicity. Data are presented as mean ± SD; n = 6 per group. ns, not significant.

**Fig S7 Histological evaluation of major organs after different treatments.** Representative H&E staining images of major organs, including the heart, liver, spleen, lung, and kidney, from mice in the Normal, CS-RB, 9,10-DiOH, and CS-RB/9,10-DiOH groups. No obvious pathological abnormalities, inflammatory infiltration, tissue necrosis, or structural damage were observed in the major organs after CS-RB/9,10-DiOH treatment, indicating good systemic biosafety of the hydrogel formulation (Scale bar = 200 μm).

**Fig S8** **Serum cytokine levels showed no significant differences among PBS, CS-RB, 9,10-DiOH, and CS-RB/9,10-DiOH groups, indicating that intra-articular administration of CS-RB/9,10-DiOH did not induce obvious systemic inflammatory responses.**

**Supplementary Tables for 9,10-DiOH Annotation and Candidate Evaluation**

**Table S1. LC-MS/MS annotation evidence for 9,10-dihydroxystearic acid (9,10-DiOH) in the two lipid formulations.**

| **Item** | **Linwa oil (Oviductus Ranae)** | **Peach kernel (Persicae Semen)** |
| --- | --- | --- |
| Compound | 9,10-Dihydroxystearic acid | 9,10-Dihydroxystearic acid |
| Feature ID | FT2403NR | FT1780NR |
| Chemical formula | C18H36O4 | C18H36O4 |
| Adducts | M-H, M+Na | M-H |
| Theoretical m/z | 315.25408 | 315.25408 |
| Measured m/z | 315.25364 | 315.25383 |
| Mass error (ppm) | -1.3957 | -0.7930 |
| Retention time (min) | 30.527 | 30.526 |
| Ion mode | NEG | NEG |
| Score | 54.77 | 70.71 |
| Fragmentation score | 0 | 75.56 |
| Fragment ions | Not detected | 297.2431, 315.2536 |
| InChIKey | VACHUYIREGFMSP-UHFFFAOYSA-N | VACHUYIREGFMSP-UHFFFAOYSA-N |
| CAS | 120-87-6 | 120-87-6 |
| Database identifiers | HMDB0302281; METLIN 35467; LipidMaps LMFA02000142; KEGG C19622; PubChem 89377 | HMDB0302281; METLIN 35467; LipidMaps LMFA02000142; KEGG C19622; PubChem 89377 |
| Chemical class | Fatty Acyls | Fatty Acyls |
| Annotation level | level2 | level1 |

Note: Linwa oil refers to Oviductus Ranae, and peach kernel refers to Persicae Semen. Compound annotation was performed using the LuMet-TCM database based on accurate MS1 mass, retention time, and MS/MS information where available. Peak area values are presented only as semi-quantitative MS response values for the same annotated feature and were not used for cross-compound abundance ranking.

**Table S2. Semi-quantitative MS response and candidate-compound evaluation of the chromatographic features assigned to 9,10-DiOH.**

| **Item** | **Linwa oil ( Oviductus Ranae)** | **Peach kernel ( Persicae Semen)** |
| --- | --- | --- |
| Feature ID | FT2403NR | FT1780NR |
| Annotated compound | 9,10-Dihydroxystearic acid | 9,10-Dihydroxystearic acid |
| Peak area / MS response | 6,794,556.61 | 37,082,621 |
| Peak area ratio (%) | 0.0613 | 0.0777 |
| Candidate-compound list | Checked | Checked |
| Alternative candidate(s) | None | None |
| Comment | No higher-confidence alternative candidate was identified. | No higher-confidence alternative candidate was identified. |

Note: Peak area values are presented only as semi-quantitative MS response values for the same annotated feature in each lipid formulation. They were not used to compare the abundance of chemically different compounds because untargeted LC-MS/MS peak areas are influenced by compound-specific ionization efficiency and MS response.

**Table S3. Shared fatty-acyl compounds detected in both lipid formulations by LuMet-TCM database-assisted annotation.**

| **No.** | **Compound** | **Formula** | **CAS** | **Level in Linwa oil** | **Level in peach kernel** |
| --- | --- | --- | --- | --- | --- |
| 1 | 2-Hydroxypalmitic acid | C16H32O3 | 764-67-0 | level1 | level1 |
| 2 | 3-oxo-2-pentylcyclopentaneacetic acid | C12H20O3 | 98674-52-3 | level2 | level1 |
| 3 | 9,10-Dihydroxystearic acid | C18H36O4 | 120-87-6 | level2 | level1 |
| 4 | Paullinic acid | C20H38O2 | 17735-94-3 | level1 | level2 |
| 5 | Petroselinic acid | C18H34O2 | 593-39-5 | level1 | level1 |

Note: All listed compounds belong to Fatty Acyls. This table is intended to support shared-component annotation rather than abundance ranking among different compounds.
